# Asymmetric RBBP7 regulates the first cell fate decision of early mammalian embryos

**DOI:** 10.1038/s41421-026-00912-6

**Published:** 2026-08-18

**Authors:** Lin-Fang Ju, Xiao Han, Danru Zhang, Heng-Ji Xu, Shaokang Jia, Meng-Xia Liu, Yanchen Liu, Yu-Ting Cai, Yu-Sheng Chen, Chun-Chun Gao, Yong-Liang Zhao, Yajing Hao, Xiu-Jie Wang, Dangsheng Li, Xiang Zhou, Ying Yang, Jianyong Han, Yun-Gui Yang

**Affiliations:** 1 Beijing Key Laboratory of Intelligent Governance and Application of Biological Big Data, China National Center for Bioinformation, Beijing, China; 2 Beijing Institute of Genomics, Chinese Academy of Sciences, Beijing, China; 3 University of Chinese Academy of Sciences, Beijing, China; 4 Sino-Danish College, University of Chinese Academy of Sciences, Beijing, China; 5 State Key Laboratory of Animal Biotech Breeding, College of Biological Sciences, China Agricultural University, Beijing, China; 6 Key Laboratory of Biomedical Polymers of Ministry of Education, College of Chemistry and Molecular Sciences, Wuhan University, Wuhan, Hubei, China; 7 School of Life Sciences and Medicine, Shandong University of Technology, Zibo, Shandong, China; 8 Institute of Genetics and Developmental Biology, Chinese Academy of Sciences, Beijing, China; 9 State Key Laboratory of Molecular Biology, State Key Laboratory of Cell Biology, Shanghai Key Laboratory of Molecular Andrology, Shanghai Institute of Biochemistry and Cell Biology, Center for Excellence in Molecular Cell Science, Chinese Academy of Sciences, University of Chinese Academy of Sciences, Shanghai, China

**Keywords:** Cell biology, Developmental biology

## Abstract

Asymmetric transcription of noncoding RNA *LincGET* is currently recognized as the earliest event regulating the first cell fate decision in mammalian embryogenesis. However, whether key protein factors modulate this process remains elusive. Here, we identify RBBP7 as the earliest protein factor regulating developmental cell fate in mammals. Loss of RBBP7 drives cells towards ICM lineage. In mouse late 2-cell embryos, unequal translation of *Rbbp7* contributes to its asymmetric protein distribution, which subsequently induces inversed asymmetric histone acetylation H3K9ac by interaction with HDAC1, thereby promoting cell differentiation. Interestingly, RBBP7 and *LincGET* exhibit a consistent asymmetric tendency but direct different cell fates; depletion or overexpression of both *Rbbp7* and *LincGET* restored the cell fate bias, suggesting a coordinated regulatory mechanism during initial lineage specification. In summary, our study reveals RBBP7 as a new protein factor and elucidates its role in the first cell fate decision.

## Introduction

The differentiation of a totipotent fertilized cell into an embryo with distinct cell lineages represents a central and fundamental question in developmental biology. In mammalian embryos, such as those of mice, cells have traditionally been deemed to possess identical developmental potential until the compacted morula stage (8- to 16-cell), owing to their pronounced plasticity and morphological consistency^1,2^. The morula stage, where cells are allocated to inner or outer positions, is thus widely regarded as the earliest cell symmetry-breaking and cell fate decision in mouse embryonic development. At this stage, inner cells inside embryos develop into inner cell mass (ICM), which subsequentially gives rise to fetus and yolk sac, whereas outer cells differentiate into trophectoderm (TE), the precursor of the placenta^2,3^. A fundamental question that remains to be fully elucidated is whether heterogeneous molecules present at even earlier stages actively orchestrate the initial emergence of cell polarity and divergent developmental trajectories.

Accumulating evidence identified that such asymmetric molecules emerge as early as the 4-cell or even 2-cell stages, indicating that the first cell symmetry-breaking and cell fate decisions occur much earlier than the previously reported morula stage^4–12^. In 4-cell mouse embryos, blastomeres with slower OCT4 kinetics, prolonged SOX2 DNA binding, and higher expression of factors like *Sox21*/*Carm1*/*Prmd14*, are biased toward the ICM fate^4–6,9,10^. Conversely, other blastomeres tend to adopt a TE fate. Remarkably, at earlier stage, 2-cell stage, the asymmetric expression of noncoding RNA *LincGET* and heterogenous accumulation of CARM1-positive paraspeckles in sister blastomeres have been implicated in the earliest cell fate bias following the first embryonic cleavage division. Blastomeres with lower *LincGET* levels and fewer CARM1-paraspeckles are predisposed to a TE cell fate, whereas those with higher levels contribute preferentially to the ICM^11,12^. Recently, a global proteomic study of several hundred asymmetrically distributed proteins which might function in cell fate bias at the 2-cell stage indicates that asymmetry is a more common phenomenon than previously appreciated^13^.

Chromatin remodeling is a key event regulating cell fate decisions by modulating chromatin accessibility^14,15^. For example, CARM1-mediated histone H3 arginine methylation (H3R26me and H3R26me2), facilitated by *LincGET*, is a known driver of the initial cell fate bias to ICM cells^4,11,12^. In addition, the nucleosome remodeling and deacetylase (NuRD) is a complex composed of multiple proteins such as MTA1, HDAC1 and RBBP7. NuRD complex promotes transcriptional heterogeneity in pluripotency genes within embryonic stem cells (ESCs), thereby reinforcing cell fate specificity of ICM^16,17^. Importantly, the NuRD complex also plays roles in cell differentiation and embryonic development, especially in the cell lineage commitments of neural stem cells and neural precursors^16–20^. As one component of the NuRD complex and the main histone deacetylase, HDAC1 catalyzes the deacetylation of lysine residues on the N-terminal part of the core histones and exhibits functional regulation in early mouse development^21,22^. HDAC1 is also related to the embryonic transcriptionally repressive regulation of histone H4 deacetylation in 2-cell embryos^23^. RBBP7 (Retinoblastoma binding protein 7) is another component of the NuRD complex, and has been reported to promote transcriptional repression by histone deacetylation and nucleosome remodeling^16,18,22^. Given its role as a chromatin remodeler and a core component of NuRD complex, whether RBBP7 plays a functional role in early embryonic development and cell fate determination is also a question well worth studying.

In this study, we identified RBBP7 as a critical protein, asymmetrically distributed regulator at the late 2-cell stage. We demonstrate that RBBP7 protein levels are translationally regulated, leading to an imbalance between sister blastomeres. In mouse late 2-cell embryos, this asymmetric RBBP7 interacts with HDAC1 to catalyze H3K9ac deacetylation, thereby directing the blastomeres toward distinct cell differentiations pathways. Moreover, we find that RBBP7 protein and *LincGET* RNA levels are inversely correlated between blastomeres. While knockdown of one does not affect the other, simultaneous perturbation of both *Rbbp7* and *LincGET*, via double knockdown or co-overexpression, partially rescues the cell fate deviation, indicating a cooperative role in early cell symmetry-breaking and cell fate determination. To conclusively profile translational asymmetry, we developed a single-cell translatome sequencing (scpRibo-seq) method on the basis of RiboLace and LiRibo-seq^24,25^, which confirmed the asymmetric translation of *Rbbp7* in individual 2-cell blastomeres. Collectively, our work identifies RBBP7 as the earliest known asymmetric protein factor and delineates its cooperative mechanism with *LincGET* in initiating the first cell fate decision in mammalian embryogenesis.

## Results

### *Rbbp7* deficiency biases cells to ICM fate

**Fig. 1:**
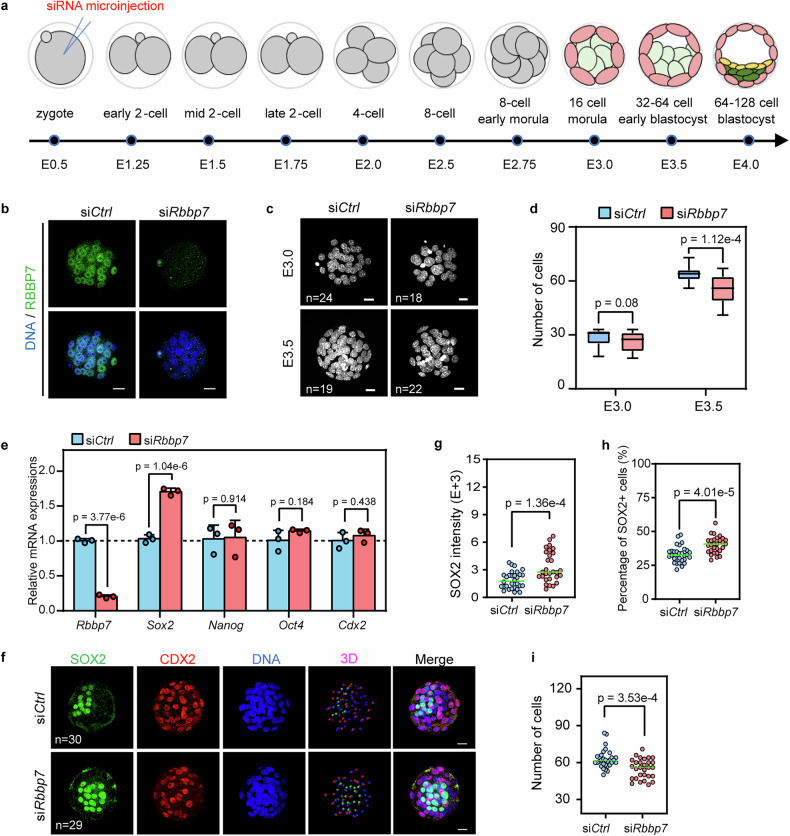
*Rbbp7* inhibits embryonic differentiation to ICM fate. **a** Scheme of the *Rbbp7* knockdown experiment. Zygotes were injected with control or *Rbbp7* siRNA. Embryos were collected at different stages for subsequent experiments. The inner cells in green indicate ICM cells, and the outer cells in red contribute to TE cells. **b** Immunofluorescence staining of RBBP7 in E3.0 si*Ctrl* and si*Rbbp7* embryos. Scale bar, 20 μm. **c** Confocal imaging of DNA stained by DAPI in si*Ctrl* and si*Rbbp7* embryos at E3.0 and E3.5 stages (E3.0 si*Ctrl* embryos: *n* = 24; E3.0 si*Rbbp7* embryos: *n* = 18; E3.5 si*Ctrl* embryos: *n* = 19; E3.5 si*Rbbp7* embryos: *n* = 22). Scale bar, 20 μm. **d** Number of cells in si*Ctrl* and si*Rbbp7* embryos at E3.0 and E3.5 (Student’s *t*-test). **e** Barplot of mRNA fold changes of *Rbbp7*, *Sox2*, *Nanog*, *Oct4*, and *Cdx2* determined by RT-qPCR (*n* = 60 for three replicates; Student’s *t*-test). Among them, *Sox2*, *Nanog*, and *Oct4* are ICM markers, *Cdx2* is a TE marker. The fold changes of all genes were normalized to the expression level of *18S* rRNA. Error bars represent SEM. **f** 3D confocal images analyzing the co-immunofluorescence analysis for SOX2 and CDX2 in E3.5 si*Ctrl* and si*Rbbp7* embryos. Scale bar, 20 μm. (si*Ctrl* embryos: *n* = 30; si*Rbbp7* embryos: *n* = 29). **g**–**i** Quantification of percentage of the fluorescent intensity of SOX2 (**g**), percentage of SOX2^+^ cells (**h**), and cell numbers (**i**) by 3D reconstruction in **f**. The *P* values were determined by the Student’s *t*-test. SOX2^+^ cells were defined as those with higher fluorescence for SOX2 than CDX2, and vice versa. Double-positive (SOX2^+^CDX2^+^) cells were excluded from this analysis.

To test whether RBBP7 regulates cell fate of early embryos, we depleted *Rbbp7* by microinjecting the specific *Rbbp7* siRNA into zygotes (Fig. 1a) and found that RBBP7 proteins were markedly depleted at morula (Embryo day 3.0 after fertilization; E3.0) stage (Fig. 1b). After *Rbbp7* depletion, we documented the morphological changes of control and *Rbbp7* depleted embryos at different developmental timepoints, including 2-cell stage, 4-8 cell stage, morula stage, and blastocyst stage. Compared to control embryos, the images acquired by stereomicroscope failed to show any obvious difference in the morphology of *Rbbp7*-deficienct embryos from 2-cell to blastocyst stages, and meanwhile, the blastocyst ratio was not affected by the decreased *Rbbp7* (Supplementary Fig. S1a, b), which coincides with the previous results^21,26^. However, even though no significant influence on E3.0 embryos was observed upon *Rbbp7* depletion, the cell number of blastocyst embryos (E3.5) was significantly reduced (Fig. 1c, d; Supplementary Table S1).

We then collected the control and *Rbbp7* deficient embryos at E3.5 stage and performed real-time quantitative PCR (RT-qPCR) to analyze the changes of classical lineage markers. Interestingly, the expression of *Sox2*, as an early and specific ICM marker at this stage^27^, was significantly increased, while other markers including *Nanog*, *Oct4*, or *Cdx2* were not significantly affected (Fig. 1e), implying that *Rbbp7* deficiency may influence the consequent ICM fate by regulating expression of *Sox2*. This observation was further confirmed by immunofluorescence staining of SOX2, showing that depletion of *Rbbp7* led to a significant increase in SOX2 intensity and percentage of SOX2^+^ cells (Fig. 1f−i; Supplementary Table S2).

### Asymmetric RBBP7 in late 2-cell embryos

**Fig. 2:**
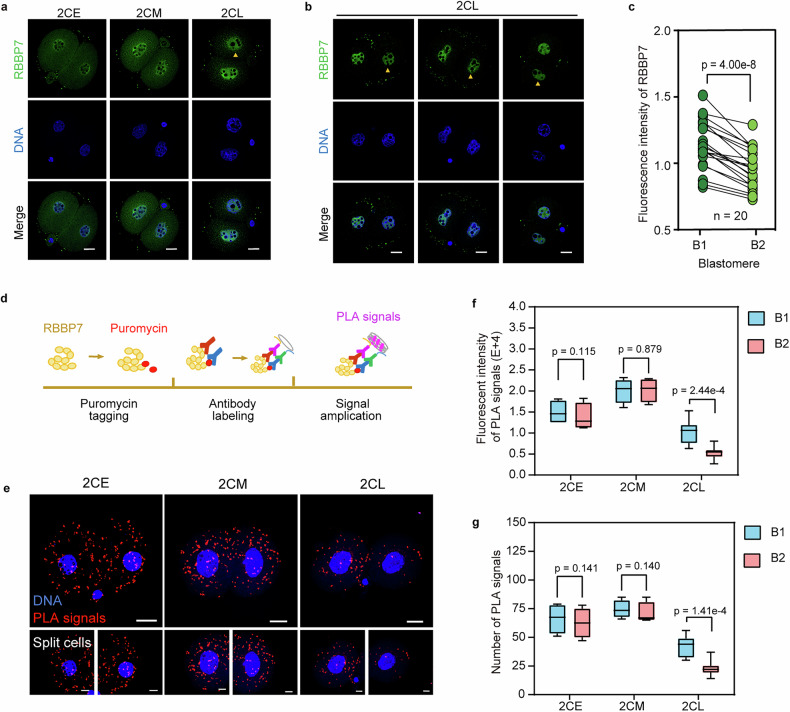
Unequal translation of *Rbbp7* leads to an asymmetric protein expression of RBBP7. **a** Immunofluorescent staining of RBBP7 in early (2CE), mid (2CM), and late (2CL) 2-cell embryos. The yellow triangles indicate blastomeres with relatively less RBBP7. Scale bar, 20 μm. **b** Immunofluorescent staining of RBBP7 in early, mid, and late 2-cell embryos. Scale bar, 20 μm. **c** Quantification of the difference of RBBP7 intensity between two blastomeres in 2CL embryos of **b**. RBBP7 intensity was normalized to DNA intensity. Blastomeres are labeled as B1 and B2 according to decreasing fluorescent intensity. **d** Schemas of RBBP7 Puro-PLA experiment. Puromycin labels the synthesized proteins, and PLA signals represent the translating proteins. **e** Confocal images of RBBP7 Puro-PLA showing the translating RBBP7 in 2CE, 2CM, and 2CL embryos (2CE embryos: *n* = 4; 2CM embryos: *n* = 4; 2CL embryos: *n* = 10). Scale bar, 20 μm. **f**, **g** Quantification analysis of the number of Puro-PLA and the fluorescence of PLA by 3D reconstruction in **e**. Blastomeres are labeled as B1 and B2 according to decreasing PLA fluorescent spots/intensity in **f**, **g**.

To explore when RBBP7 begins to influence cell fate, we first examined its protein expression by immunofluorescence staining across mouse early (2CE), mid (2CM), and late (2CL) 2-cell embryos. While RBBP7 levels were uniform between sister blastomeres in 2CE and 2CM embryos, a pronounced asymmetry emerged specifically at the 2CL stage (Fig. 2a–c). To investigate the origin of this heterogeneity, we asked whether it arose from transcriptional or translational regulation. RNA fluorescence in situ hybridization (FISH) was performed and revealed no significant difference in *Rbbp7* transcript levels between sister blastomeres (Supplementary Fig. S2a). We therefore employed a puromycylation-proximity ligation assay (Puro-PLA)^28^ to directly visualize nascent RBBP7 protein synthesis, directly reporting on its active translation (Fig. 2d). We found that in 2CL embryos, both the number and intensity of PLA signals were significantly asymmetric between sister blastomeres (B1 and B2), a disparity not observed in earlier 2CE or 2CM stages (Fig. 2e−g).

Nevertheless, the total translation levels between blastomeres had no change since the global proteins labeled by puromycin did not show obvious difference between sister blastomeres throughout all 2-cell stages (Supplementary Fig. S2b). Collectively, these data demonstrate that the heterogeneous distribution of RBBP7 protein in late 2-cell embryos is a consequence of asymmetric translation of its mRNA.

### RBBP7 interacts with HDAC1 to deacetylate H3K9ac in late 2-cell embryos

**Fig. 3:**
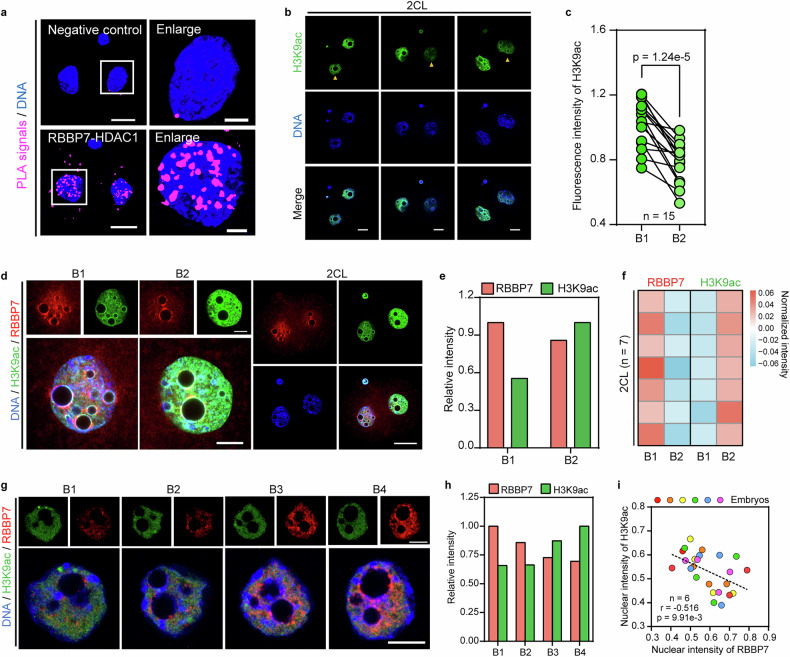
RBBP7 interacts with HDAC1 to deacetylate H3K9ac in 2CL embryos. **a** Confocal imaging of RBBP7-HDAC1 PLA in 2CL embryos. Negative control was performed with antibody against RBBP7 only. Scale bar, 20 μm (left); 5 μm (right, enlarged region). **b** Immunofluorescence staining of H3K9ac in 2CL embryos. The yellow triangles indicate blastomeres with relatively less H3K9ac. Three experimental replicates were performed. Scale bar, 20 μm. **c** Quantification of H3K9ac fluorescence intensity in blastomeres of 2CL embryos (*n* = 15). H3K9ac intensity was normalized to DNA fluorescence intensity. Blastomeres are labeled as B1 and B2 according to decreasing H3K9ac fluorescent intensity. **d** Co-IF of RBBP7 and H3K9ac in 2CL embryos. Scale bar, 10 μm (left) and 30 μm (right). **e** The intensity analysis of embryo (**d**) shows that RBBP7 and H3K9ac are inversely correlated in blastomeres. RBBP7/H3K9ac intensity was normalized to DNA fluorescence intensity. **f** Quantification of RBBP7-H3K9ac co-IF (*n* = 7). RBBP7/H3K9ac intensity was normalized to DNA fluorescence intensity. **g** Co**-**IF of RBBP7 and H3K9ac in 4-cell embryos. Scale bar, 10 μm. **h** The intensity analysis of embryo (**g**) shows that RBBP7 and H3K9ac are inversely correlated in blastomeres. RBBP7/H3K9ac intensity was normalized to DNA fluorescence intensity. **i** Quantification analysis of RBBP7-H3K9ac co-IF in 4-cell embryos (*n* = 6; Spearman’s correlation coefficient). Blastomeres are labeled as B1 to B4 according to decreasing RBBP7 fluorescent intensity in **d–h**.

To elucidate how asymmetric RBBP7 regulates cell fate in 2-cell embryos, we first sought to identify its protein interactors. We performed RBBP7 immunoprecipitation in combination with mass spectrometry (IP-MS) in mouse embryonic stem cells (mESCs). Notably, this analysis revealed significant enrichment of HDAC1 and other core components of NuRD complex, including RBBP4, HDAC2, CHD4, GATAD2A/B, MTA1/2/3^16^, in the RBBP7 immunoprecipitants (Supplementary Fig. S3a and Table S3). In contrast, the main subunits of other known RBBP7-containing complexes (including SIN3, CoREST, PRC2, HAT1, NURF, and Ash1 complexes)^29–32^ were not significantly enriched, suggesting that RBBP7 may exert deacetylation function through NuRD complex in early embryos. The PLA of RBBP7–HDAC1 showed scattered interaction signals in nucleus of 2CL embryos (Fig. 3a), indicating that RBBP7 indeed interacts with HDAC1. Although the distribution of HDAC1 has been reported to become heterogenous at later morula and blastocyst stages of mouse embryos^33^, it remained symmetric distribution between sister blastomeres throughout the 2-cell stage (Supplementary Fig. S3b). We therefore propose that the asymmetry of RBBP7, rather than HDAC1, might drive the heterogeneity in histone acetylation.

To investigate which histone mark is targeted by RBBP7-mediated deacetylation, we performed immunofluorescence staining of potential histone acetylated modifications to determine whether differential histone acetylation exists in 2CL embryos. Intriguingly, H3K9ac manifested significant cell-cell differences in 2CL embryos (Fig. 3b, c), while other acetylation marks, including H3K18ac, H3K27ac, H4K5ac, and H4K12ac, displayed similar acetylation levels between sister blastomeres (Supplementary Fig. S3c). Of note, we only observed asymmetric H3K9ac in 2CL embryos rather than in 2CE or 2CM embryos (Supplementary Fig. S3d), consistent with the asymmetric status of RBBP7 protein at 2-cell stage (Fig. 2a). Furthermore, the interaction between RBBP7 and H3K9ac was demonstrated by PLA in 2CL embryos (Supplementary Fig. S3e). Importantly, through co-immunofluorescence staining of RBBP7 and H3K9ac, we observed a total opposite tendency in the asymmetry of H3K9ac and RBBP7 in 2CL embryos (Fig. 3d−f): blastomeres with more RBBP7 exhibited less H3K9ac, and vice versa. Similar observations were also observed in 4-cell embryos (Fig. 3g−i). All these results identify asymmetric RBBP7 as a potential driver of heterogenous H3K9ac deacetylation in 2-cell mouse embryos.

### Asymmetric H3K9ac induced by RBBP7 correlates with differential cell differentiation

Dynamic epigenetic modifications regulate the early mammalian embryos through inducing reprogramming events^4,34^. Previous studies have established that H3K9ac predominantly distributes in ICM cells of mouse blastocyst^33^, and preferentially activates ICM-associated genes in stem cells^33,35^. This provides a mechanistic link between H3K9ac enrichment and ICM fate specification.

**Fig. 4:**
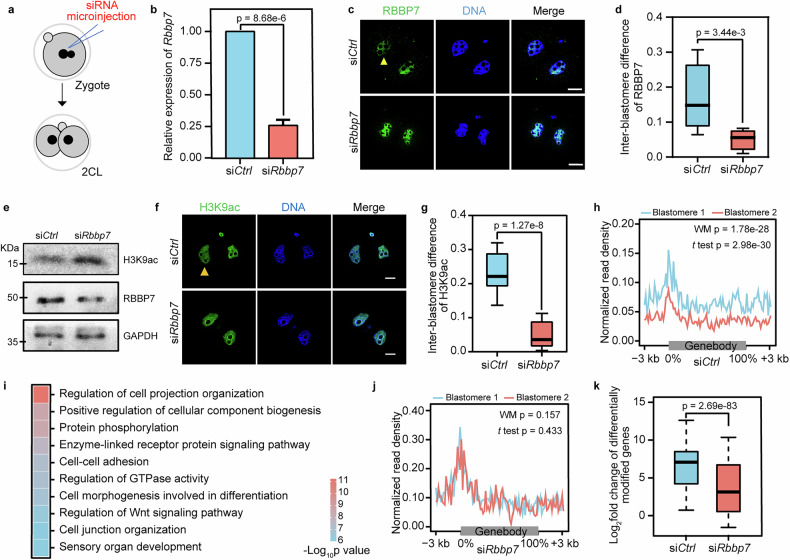
Differential H3K9ac induced by RBBP7 leads to asymmetric cell differentiation. **a** Schemas of the *Rbbp7* knockdown experiment. Zygotes were injected with control or *Rbbp7* siRNA. Embryos were collected at 2CL stage. **b** quantification of *Rbbp7* knockdown efficiency in 2CL determined by RT-qPCR (*n* = 60 for three replicates; Student’s *t*-test). Error bars represent SEM. **c**, **d** Immunofluorescence staining (**c**) and quantification (**d**) of RBBP7 in 2CL si*Ctrl* or si*Rbbp7* embryos (Student’s *t*-test). The yellow triangle indicates blastomere with relatively less RBBP7. RBBP7 intensity was normalized to DNA fluorescence intensity. Scale bar, 20 μm. **e** Western blotting showing the expression of H3K9ac, RBBP7 in 2CL si*Ctrl* and si*Rbbp7* embryos. GAPDH protein serves as protein loading control. **f**, **g** Immunofluorescence staining (**f**) and quantification (**g**) of H3K9ac in 2CL si*Ctrl* or si*Rbbp7* embryos (Student’s *t*-test). The yellow triangle indicates blastomere with relatively less H3K9ac. H3K9ac intensity was normalized to DNA fluorescence intensity. Scale bar, 20 μm. **h** Metagene profiles of normalized H3K9ac read density in two blastomeres of si*Ctrl*_1 embryo. **i** GO enrichment analysis of differential H3K9ac modified genes between blastomeres from si*Ctrl*_1 embryo. **j** Metagene profiles of normalized H3K9ac read density in two blastomeres of si*Rbbp7*_1 embryo (WM refers to two-sided Wilcoxon and Mann-Whitney test, *t*-test refers to two-sided paired Student’s *t*-test). **k** Boxplot showing the abundance changes of H3K9ac signals of differential H3K9ac modified genes between blastomeres from si*Ctrl* embryos in si*Ctrl* and si*Rbbp7* samples (Student’s *t*-test).

To investigate whether this asymmetric RBBP7 regulates cell fate through its inducing differential H3K9ac, we collected *Rbpp7* deficient and control embryos at 2CL stage (Fig. 4a, b). We observed that *Rbbp7* deficiency destroyed asymmetry of RBBP7 (Fig. 4c, d), which further led to globally increased abundance of H3K9ac and significantly diminished asymmetry of H3K9ac (Fig. 4e−g). These results demonstrate the regulation of asymmetric RBBP7 on differential H3K9ac.

Next, we performed single-cell cleavage under targets and tagmentation (scCUT&Tag) of H3K9ac in the presence or absence of *Rbbp7* at 2CL stage to analyze the influence of RBBP7-H3K9ac on cell fates (See Methods). We confirmed that samples exhibited normal H3K9ac reads distribution and high correlations between biological replicates (Supplementary Fig. S4a, b and Table S4). Importantly, in control embryos (si*Ctrl*), the distribution of H3K9ac of si*Ctrl* exhibited significant cell-cell difference in read density (Fig. 4h; Supplementary Fig. S4c, d). Genes exhibiting differential H3K9ac enrichment between sister blastomeres were enriched for functions related to cell differentiation (Fig. 4i; Supplementary Fig. S4e and Table S5), suggesting that asymmetric H3K9ac in 2-cell embryos might influence subsequent cell differentiation.

We further applied scCUT&Tag to *Rbbp7-*deficient embryos (si*Rbbp7*) and observed that *Rbbp7* deficiency led to an obviously decreased extent of overall difference between sister blastomeres (Fig. 4j; Supplementary Fig. S4f−j), particularly for those differentially modified genes (Fig. 4k).

Together, these results demonstrate that asymmetric RBBP7 regulates differential H3K9ac, and the known function of H3K9ac in promoting ICM gene activation provides a mechanistic basis for how the RBBP7-H3K9ac axis influences early cell fate decisions.

### RBBP7 and *LincGET* exhibit concordant asymmetry

**Fig. 5:**
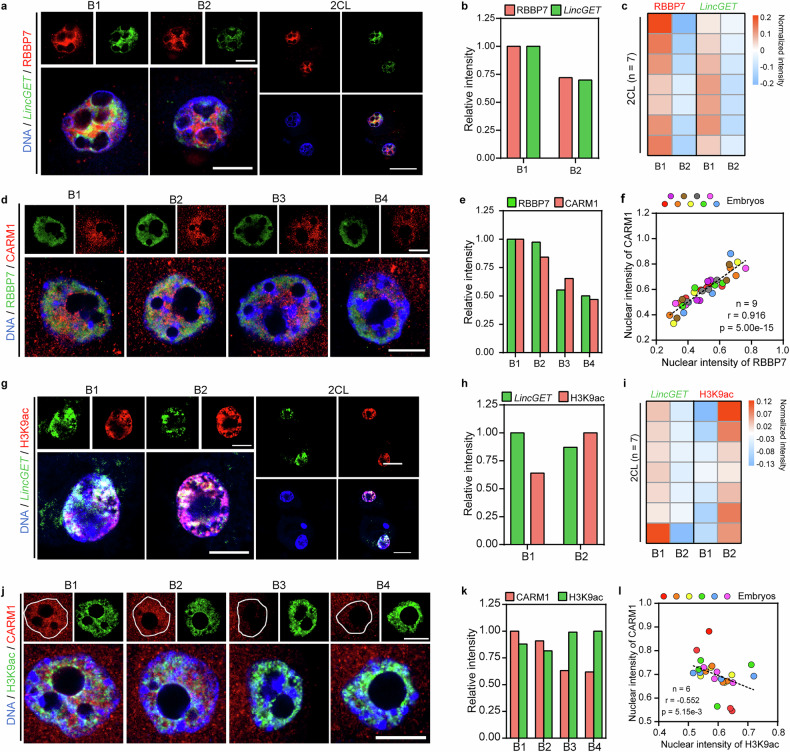
RBBP7 and *LincGET* display coordinated asymmetric patterns. **a** IF-coFISH of RBBP7 and *LincGET* in 2CL embryos. Scale bar, 10 μm (left) and 30 μm (right). **b** The intensity analysis of embryo (**a**). **c** Quantification of RBBP7 and *LincGET* IF-coFISH RBBP7/*LincGET* intensity was normalized to DNA fluorescence intensity. **d** Co-IF of RBBP7 and CARM1 in 4-cell embryos. Scale bar, 10 μm. **e** The intensity analysis of embryo (**d**). Blastomeres are labeled as B1 to B4 according to decreasing RBBP7 fluorescent intensity in **a–e**. **f** Quantification analysis of (**d**) RBBP7-CARM1 co-IF. **g** IF-coFISH of H3K9ac and *LincGET* in 2CL embryos. Scale bar (left), 10 μm; scale bar (right), 30μm**. h** The intensity analysis of embryo (**g**). **i** Quantification of IF-coFISH of H3K9ac and *LincGET*. H3K9ac/*LincGET* intensity was normalized to DNA fluorescence intensity. **j** Co**-**IF of H3K9ac and CARM1 in 4-cell embryos. Scale bar, 10 μm. **k** The intensity analysis of embryo (**j**). H3K9ac/CARM1 intensity was normalized to DNA fluorescence intensity. **l** Quantification analysis of H3K9ac-CARM1 co-IF in 4-cell embryos. Blastomeres are labeled as B1 to B4 according to decreasing CARM1 fluorescent intensity in **a–k**.

Since the asymmetric expression of *LincGET* emerges in mouse late 2-cell embryos as well^12^ and heterogenous CARM1 occurs in 4-cell embryos, we further explored the possible functional coordination between the regulatory axis of RBBP7-H3K9ac and *LincGET*/CARM1-H3R26me. Through immunofluorescence staining combined with RNA-FISH (IF-coFISH) assay, we observed that the RBBP7 protein and *LincGET* RNA displayed the same asymmetric tendency in late 2-cell embryos (Fig. 5a) with both high in one blastomere and low in another blastomere (Fig. 5b, c). Additionally, the same asymmetric tendency of RBBP7 and CARM1 were also observed in 4-cell embryos (Fig. 5d−f). Similarly, the same opposite asymmetric tendency between the distributions of H3K9ac and *LincGET* in late 2-cell embryos (Fig. 5g−i), as well as between H3K9ac and CARM1 in 4-cell embryos (Fig. 5j−l) were also observed.

Interestingly, less *LincGET* was reported to predispose early embryonic cells to TE cell fate¹², while fewer RBBP7 promote the specification of ICM cell fate (Fig. 1). This opposite functional association, despite their concordant asymmetric localization, suggests a potential interplay that warrants further investigation.

### RBBP7 and *LincGET* collaboratively regulate early cell fate bias

**Fig. 6:**
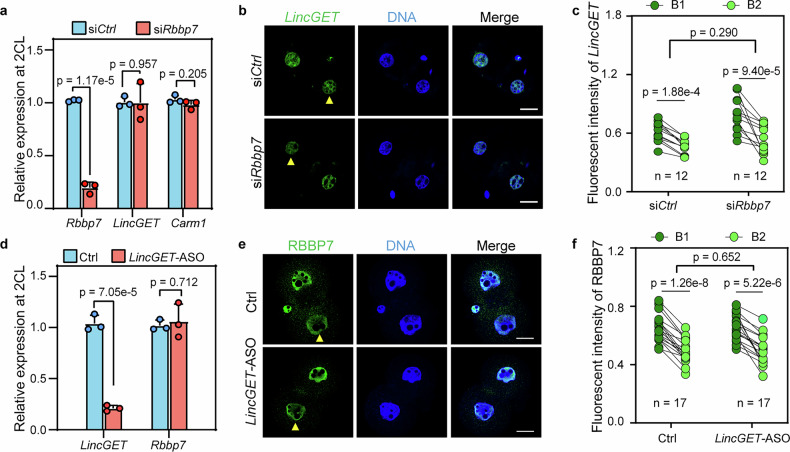
RBBP7 and LincGET asymmetries arise independently. **a** Barplots of RNA fold changes of *Rbbp7*, *LincGET*, and *Carm1* determined by RT-qPCR (*n* = 60 for three replicates; two-sided unpaired Student’s *t*-test). **b**, **c** Normalized to *18S* rRNA expression. RNA-FISH (**b**) and quantification (**c**) of *LincGET* in 2CL si*Ctrl* and si*Rbbp7* embryos. The yellow triangle indicates blastomere with relatively less *LincGET*. *LincGET* intensity was normalized to DNA fluorescence intensity. Scale bar, 20 μm. The *P* value between si*Ctrl* and si*Rbbp7* embryos was calculated by a two-sided unpaired Student’s *t*-test. **d** Barplots of RNA fold changes of *LincGET* and *Rbbp7* determined by RT-qPCR (*n* = 60 for three replicates; two-sided unpaired Student’s *t*-test). Normalized to *18S* rRNA level. **e**, **f** Immunofluorescence staining (**e**) and quantification (**f**) of RBBP7 in 2CL Ctrl or *LincGET*-ASO embryos. The yellow triangle indicates blastomere with relatively less RBBP7. Scale bar, 20 μm. The *P* value between Ctrl or *LincGET*-ASO embryos was calculated by a two-sided unpaired Student’s *t*-test. Sister blastomeres are labeled as B1 to B2 based on the decreased fluorescent intensity (**b**, **e**). *P* values in blue were determined by a one-tailed (right-sided) Wilcoxon signed-rank test, *P* values in black were determined by a one-tailed paired Student’s *t*-test.

To investigate whether asymmetric RBBP7 and *LincGET* are mutually regulated in early embryos, we first knocked down *Rbbp7* in zygotes through microinjecting siRNA into zygotes, and found that *Rbbp7* deficiency had no effect on the expression of *LincGET* or *Carm1* in either 2- or 4-cell embryos (Fig. 6a; Supplementary Fig. S5a). Meanwhile, the asymmetry of *LincGET* kept unchanged (Fig. 6b, c). Likewise, decrease of *LincGET* by its specific anti-sense oligo (ASO) did not influence the *Rbbp7* expression or RBBP7 asymmetry as well (Fig. 6d−f; Supplementary Fig. S5b). Thus, RBBP7 and *LincGET* do not appear to regulate each other’s expression or asymmetry under the conditions tested.

**Fig. 7:**
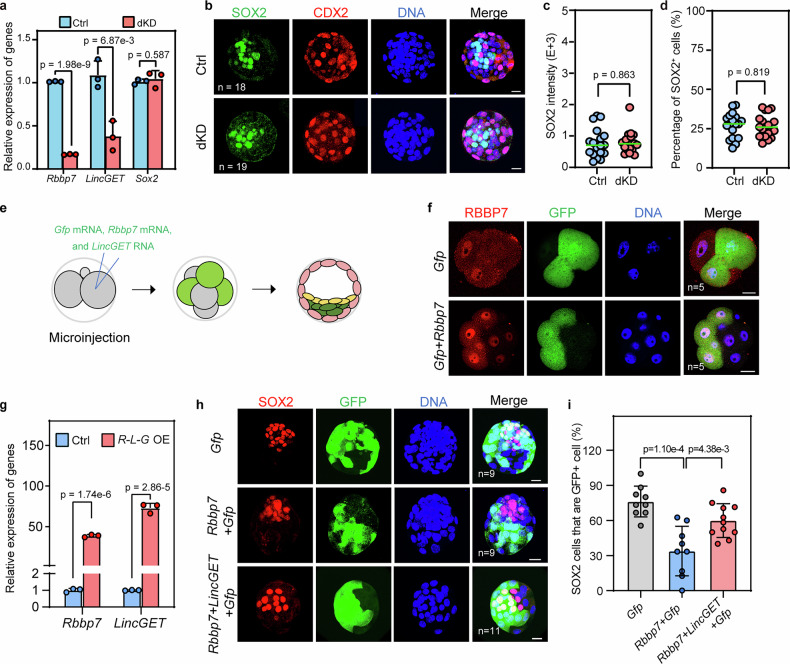
RBBP7 and *LincGET* collaboratively regulate early symmetry-breaking. **a** Barplots of mRNA fold changes of *Rbbp7*, *LincGET*, and *Sox2* determined by RT-qPCR (*n* = 60 for three replicates; Student’s *t*-test). Normalized to *18S* rRNA expression. Error bar represents SEM. **b** 3D confocal images of co-immunofluorescence analysis for SOX2 and CDX2 in E3.5 Ctrl and dKD embryos (Ctrl: *n* = 18; E3.5 dKD: *n* = 19). Scale bar, 20 μm. **c** Quantification of SOX2 intensity from the 3D reconstruction in **b**. **d** Percentage of SOX2^+^ cells quantified from the 3D reconstruction in **b**. The *p* values of (**c**, **d**) were calculated by a Student’s *t*-test. **e** Schemas of the overexpression experiment. One of the blastomeres of a 2-cell embryo was injected with either *Gfp* RNA, *Rbbp7*-*Gfp* RNAs or *Rbbp7*-*LincGET*-*Gfp* RNAs. **f** Fluorescence staining of RBBP7 and GFP in 4-8 cell embryos. Scale bar, 20 µm. **g** Barplots of relative RNA expression of *Rbbp7* and *LincGET* in Ctrl and R-L-G OE (*Rbbp7*-*LincGET*-*Gfp* overexpression) embryos (Student’s *t*-test). Normalized to *18S* rRNA expression. **h** 3D images of fluorescent SOX2 and GFP in blastocysts. Scale bar, 20 µm. **i** Barplots showing the percentages of GFP^+^ cells out of the SOX2^+^ cells in the embryos depicted in **h**. The *P* values were determined by a Student’s *t*-test.

Previous studies have established that *LincGET* knockdown biases cells toward TE fate and reduces ICM-associated gene expression, while *LincGET* overexpression promotes ICM specification^12^. To further investigate the functional interaction between RBBP7 and *LincGET*, we depleted both factors simultaneously at zygote stage and found that the double knockdown (dKD) embryos had a rescued upregulation of *Sox2* to the same level of control group (Fig. 7a). Additionally, both the SOX2 intensity and percentage of SOX2^+^ cells in dKD embryos were also back to control level, with unchanged blastocyst formation, even though the phenotype of the fewer cell numbers still exists (Fig. 7b−d; Supplementary Fig. S6a, b and Table S6).

Additionally, we simultaneously microinjected both *Gfp* and *Rbbp7* mRNAs into a single blastomere of 2-cell embryos to trace the preference of cell fate^12^ (Fig. 7e−g). Notably, when compared to embryos overexpressing *Gfp* alone, those overexpressing both *Rbbp7* and *Gfp* exhibited a significantly reduced tendency to differentiate into ICM (Fig. 7h, i; Supplementary Fig. S6c and Table S7), further proving the regulation of RBBP7 on ICM cell fate. Moreover, by comparing the results of the *Gfp* group against those of both *Rbbp7* and *LincGET* overexpressing group, we found that the fate-biased effect of *Rbbp7* was partially compensated by *LincGET* (Fig. 7h, i; Supplementary Fig. S6c). These findings illustrate the collaborative roles of signaling axes mediated by *Rbbp7* and *LincGET* in orchestrating the initial symmetry-breaking events and modulating opposing directions of cell fate differentiation in mammalian embryos.

### Single-cell translatome sequencing reveals asymmetric *Rbbp7* translation in late 2-cell embryos

Omics sequencing technologies provide powerful tools for in-depth study of cell and molecular biology and developmental biology. Although the development of single-cell proteome sequencing technique faces great challenges, several pioneering single-cell translatome techniques have achieved breakthroughs. The low-input or single-cell translatomic techniques^25,36–39,^ have been developed to reveal the stage characteristics of oocytes or early embryos, but the translatomic landscape containing translational heterogeneity in single blastomeres of whole 2-cell embryos remains to be described.

**Fig. 8:**
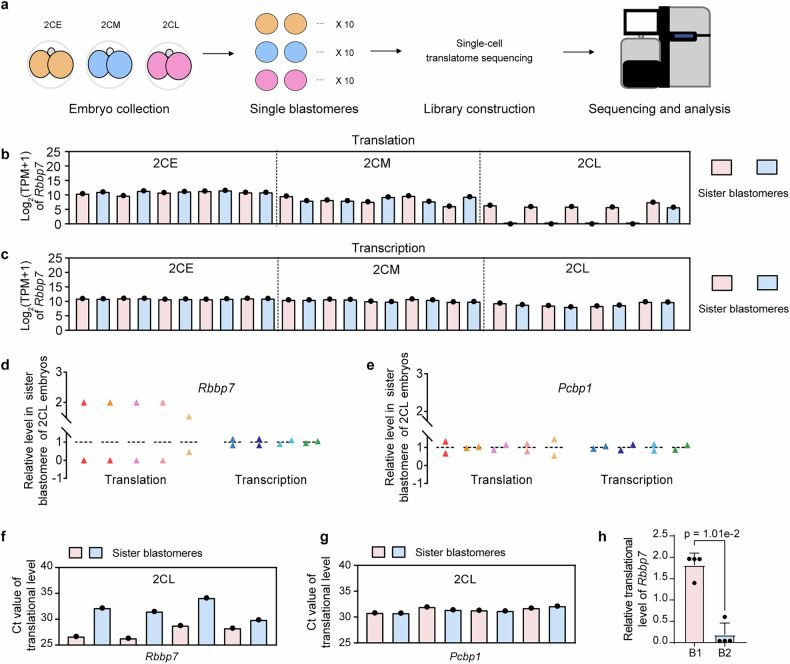
Single-cell translatome sequencing confirms asymmetric *Rbbp7* translation in late 2-cell embryos. **a** Schematic workflow showing the single blastomeres of 2-cell embryos for single-cell translatome sequencing. **b**, **c** The translational (**b**) and transcriptional (**c**) levels of *Rbbp7* in sister blastomeres of early, mid, late 2-cell embryos. Data from scpRibo-seq and scRNA-seq. **d** *Rbbp7* translational and transcriptional levels in each blastomere of 2CL embryos. **e** The translational and transcriptional levels of *Pcbp1*, as a control gene, in each blastomere of 2CL embryos. **f**, **g** Single-cell pulldown-qPCR results showing the translational distribution of *Rbbp7* (**f**) and *Pcbp1* (**g**) between blastomeres of late two-cell embryos. Ct values are used for level analysis. **h** Relative translational level of *Rbbp7* normalized to *Pcbp1*.

To obtain the translational atlas and further verify the expression of *Rbbp7* in mouse 2-cell embryos, we optimized RiboLace technique^24^ and modified it as scpRibo-seq (Supplementary Fig. S7; See Materials and methods). The single-cell translatome profiles of oocytes from scpRibo-seq exhibited normal reads distribution, high repeatability, and good translation specificity (Supplementary Fig. S7d−g). Subsequently, we applied scpRibo-seq and scRNA-seq to single sister blastomeres from early, mid, and late 2-cell embryos (Fig. 8a). The translation signals of all samples displayed high correlations among replicates and obvious stage-specificity (Supplementary Fig. S7h, i). In these data, compared to 2CE and 2CM, the translational level of *Rbbp7* in sister blastomeres of 2CL became unequal while its transcriptional level shows almost no inter-blastomere differences at whole 2-cell stages (Fig. 8b, c). *Rbbp7* exhibits obvious translational cell-cell variations in late 2-cell embryos (Fig. 8d), yet *Pcbp1*, as a control gene, showed no intercellular differences in either translational or transcriptional levels (Fig. 8e). To further strengthen the asymmetric translation of *Rbbp7*, we performed an additional validation using single cell 3P-pulldown-qPCR, which measures actively translating mRNAs from isolated sister blastomeres at the late 2-cell stage^24,38^. We observed the obvious inter-blastomere difference in the level of translating *Rbbp7* mRNA, whereas no such difference was detected for the control gene *Pcbp1* (Fig. 8f−h). These results provided key evidence supporting the conclusion that *Rbbp7* undergoes differential translation, thereby establishing the basis for its asymmetric protein levels in late 2-cell embryos.

## Discussion

So far, *LincGET* RNA and CARM1-containing paraspeckles are two early factor or complexes regulating symmetry-breaking and cell fate bias in 2-cell stage^11,12^. More recently, a global set of asymmetric proteins was identified in mouse 2-cell stage embryos, revealing that asymmetry is not a rare event but rather a common feature of early embryonic cells. In this study, we elucidate the mechanism by which the asymmetrically distributed protein RBBP7 regulates cell fate determination in early embryos. Furthermore, we provide the first explanation for the origin of such asymmetric protein distribution, demonstrating that it arises from post-transcriptional regulation — specifically, through RNA translation. Not all mRNAs are translated into proteins due to various post-transcriptional regulatory mechanisms that impact mRNAs, including their storage or degradation, post-transcriptional modifications, and interactions with RNA-related proteins. Thus, RNA translation is theoretically closer to functional protein in vivo. The approaches of single-cell quantitative microfluidics and single-cell transcriptome sequencing have been applied to preimplantation embryos to characterize the dynamic transcriptional atlas and cellular transcriptional heterogeneity. Several genes were evidenced as cell fate regulators with roles in biasing early cell fate differentiation, such as heterogenous expressions of *Prdm14* and *Sox21* in 4-cell embryos and *LincGET* in 2-cell embryos^6,10,12^. In mammalian oocytes and early embryos, the state of transcriptional silence means a dynamic and tight control on translation regulation. Despite the significant progress in dissecting the relationship between cell-cell transcriptional heterogeneity and cell fate determination, the understanding of post-transcriptional regulation especially translation control on early cell fate decision is still lacking. In this study, we employed scpRibo-seq on single blastomeres of 2-cell embryos and delineate the function of asymmetric translation of *Rbbp7* in early mammalian cell fate decision, thus facilitating to fill this gap.

NuRD complex modulates the transcriptional heterogeneity and lineage commitment by decreasing the pluripotent genes in embryonic stem cells^16,17^, and moreover, regulates cell differentiation and embryonic development^16,18–20^. RBBP7 is a key component of the NuRD complex, and has been reported to promote transcriptional repression by histone deacetylation and nucleosome remodeling^16,18,22^. Previous findings showed that knockdown of *Rbbp7* did not influence the blastocyst formation^21,26^, whereas double depletion of *Rbbp7* and its highly homologous gene, *Rbbp4*, results in the disruption of lineage specification and chromatin structure, accompanied by a dramatic increase in H3.3, H3K27ac, and 5mC abundance during mouse morula-to-blastocyst transition^21,26^. Interestingly, only *Rbbp7* knockdown leads to normal blastocyst formation but with less cell numbers, whereas double knockdown of both *Rbbp7* and *Rbbp4* resulted in phenotypes resembling those observed in the depletion of both *Hdac1* and *Hdac2*, characterized by severe developmental arrest due to the decreased cell proliferation and enhanced cell apoptosis^21,40^. Therefore, we hypothesize that the effect of *Rbbp7* depletion during embryonic development might be partially compensated by *Rbbp4*, while the reduction in cell numbers is likely attributed to the reduced cell proliferation and increased cell apoptosis.

As a core subunit of the NuRD complex, RBBP7 function is highly sensitive to subtle reductions in its protein abundance. Accumulating structural and biochemical evidence demonstrates that intact subunit stoichiometry is indispensable for normal NuRD activity. RBBP7 and RBBP4 are dynamically integrated core subunits whose incorporation is vulnerable to expression perturbations^41^. Even mild reduction of these stoichiometric subunits can compromise complex assembly, stability and genomic targeting, thereby leading to prominent functional defects. This type of dosage sensitivity has also been well documented in other chromatin regulatory complexes^42^. This also explains why partial knockdown of RBBP7 in our study is sufficient to impair the function of the NuRD complex.

**Fig. 9:**
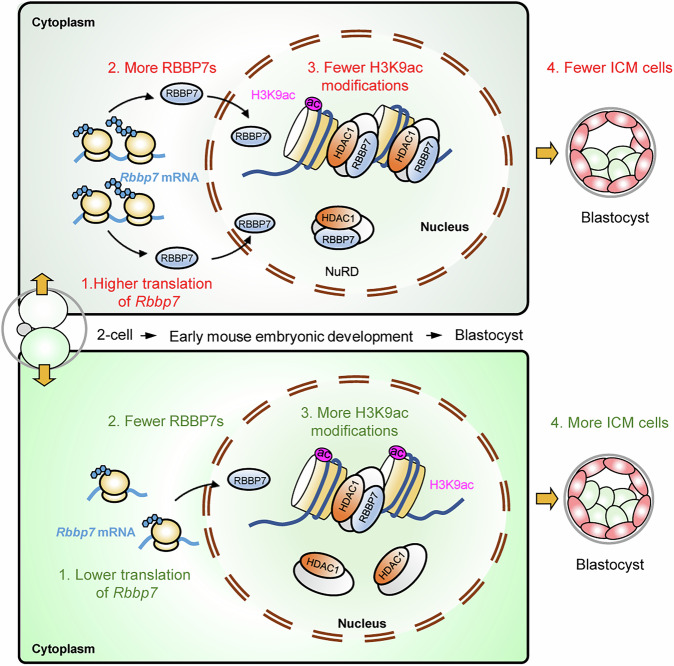
Schematic models of *Rbbp7*-H3K9ac-driven ICM cell fate bias. RBBP7 and HDAC1 as two components of NuRD complex, deacetylate H3K9ac. The blastomere with lower *Rbbp7* translation but more H3K9ac is destined for more ICM cells.

Except for H3K27ac and H4K5ac^21,26^, RBBP7-HDAC1 also deacetylates the H3K4ac, H3K8ac, H3K12ac, and H3K16ac, ensuring the normal positioning and function of the chromosomal process complex during the meiotic maturation of mouse oocytes^43^. Moreover, as a core component of NuRD, chromodomain helicase DNA binding protein 3 (CHD3) has been shown to co-localize with HDAC1 and H3K9ac in K562 and LNCaP cell lines^44^. Our finding verifies the direct regulation of RBBP7-HDAC1 on H3K9ac. Given that the differential H3K9ac pathway is associated with cell differentiation (Fig. 4i), we hypothesize that H3K9ac might regulate the downstream expression of specific genes that determine cell fate during the 2-cell stage. The detailed mechanism of differential H3K9ac regulating cell fate of 2-cell stage needs to be further explored in the future. Of importance, H3K9ac preferentially activates ICM-associated genes (including *Sox2*, *Nanog*, *Oct4*, *Rex1*, *etc*.) in mESCs derived from ICM cells, and also upregulates *Sox2* and *Rex1* in trophectoderm stem cells derived from TE cells^35^. In this study, we found that depletion of *Rbbp7* results in an increased number of SOX2-positive cells (ICM cells) and higher levels of SOX2 expressions. We propose a model depicting the regulation mode guided by the *Rbbp7*-H3K9ac axis: the asymmetric RBBP7 and unequal H3K9ac modifications specifically regulate the fate of ICM cells in early embryos (Fig. 9).

Epigenetic reprogramming is a prerequisite for preimplantation embryo development^34,45^. Previous studies proved that histone methylation has critical impacts on cell fate decision. The dynamic patterns of H3K4me3 and H3K27me3 represent the epigenetic features in activating the genome during mouse ZGA^46–48^. The heterogeneity of H3R26me at mouse 4-cell stage was shown to be the earliest epigenetic modification participating in cell fate choice^4^. Especially, both *LincGET* coupled with CARM1 and CARM1-containning paraspeckles increase H3R26me level and upregulate ICM-specific genes, biasing the cell fate towards ICM^4,11^. Here we reported a translationally controlled mechanism for early symmetry-breaking for regulating ICM/TE cell fate decision through differential histone acetylation H3K9ac in 2-cell embryos, advancing our understanding of the epigenetic regulation in cell fate to 2-cell stage from previously reported 4-cell stage^4,11^.

An intriguing observation from our study is that RBBP7 and *LincGET* exhibit concordant asymmetry in the same blastomere yet are associated with opposing cell fates — fewer RBBP7 promotes ICM fate, while less *LincGET* predisposes cells to TE fate^12^. This raises the possibility of a functional interaction between these two regulatory axes in coordinating symmetry-breaking and lineage specification. Future studies will be needed to dissect the molecular basis of this potential crosstalk.

Intriguingly, we offered a new symmetric-breaking regulatory axis of *Rbbp7*-H3K9ac, which acts cooperatively with the previously reported *LincGET*-CARM1-H3R26me axis to bias the early cell fate determination in mouse 2-cell embryos. We propose that this reflects a temporal and functional division of labor in early lineage specification. According to previous studies, *LincGET* functions as a lineage force that promotes ICM specification from the 2-cell to 8-cell stage^12^, while our data show that RBBP7 acts during the 2-cell to 4-cell stage to oppose ICM fate (less RBBP7 biases toward ICM; more RBBP7 biases toward TE). Thus, rather than directly antagonizing each other at the same time, these two factors may operate in overlapping but distinct temporal windows, with RBBP7 functioning as an early brake on ICM commitment, and *LincGET* subsequently promoting and stabilizing ICM fate once cells begin to diverge.

Consistent with this temporal regulatory pattern, our E3.5 lineage marker analysis further supports the early cell fate modulation by RBBP7. *Rbbp7* depletion specifically upregulated the early and ICM-specific marker *Sox2*, while other core lineage markers, including *Nanog*, *Oct4*, and *Cdx2*, remained unaltered (Fig. 1e). Further validation showed that the early TE marker *Id2* was reduced, whereas *Esrrb* exhibited no significant changes in *Rbbp7*-deficient embryos (Supplementary Fig. S1d). Since *Sox2* and *Id2* represent earlier lineage specifiers that are more sensitive to embryonic perturbation than late-stage markers^27^, these results demonstrate that RBBP7 predominantly modulates the earlier cell fate decisions, with minimal effects on later lineage.

This temporal model is consistent with the concept of “lineage strength” proposed in previous work^8^, in which early blastomeres possess a flexible, yet predisposed, lineage trajectory prior to morphological differentiation. In this framework, RBBP7 and *LincGET* can be viewed as opposing lineage forces that collectively regulate the timing and direction of the first cell fate decision, preserving cell plasticity during the 2- to 4-cell stages. After this stage, there might be other factors involved, such as heterogeneous HDAC1 which starts to play a role from the 8-cell stage^33^, and regulating the cell fate in later stages through cooperating with CARM1 and *LincGET*. Moreover, *Rbbp7* is a maternally expressed gene, and the differential expression of *LincGET* in late 2-cell embryos has been hypothesized to result from the inconsistencies in zygotic gene activation (ZGA) between sister blastomeres^13^. Therefore, there may be some specific translation regulators, acting as downstream effector of the inconsistent ZGA, and modulating the differential translation of *Rbbp7* at this stage. Additionally, it has been reported that the RNA *N*^6^-methyladenosine (m^6^A) modifications exhibit an asymmetrical distribution pattern in mouse 2-cell embryos^49^, which is more likely to trigger the asymmetric translation of *Rbbp7*.

### Limitations of this study

This study has several limitations. First, although we identify asymmetric translation as the origin of RBBP7 heterogeneity, the underlying mechanistic basis, which may involve RNA modifications, remains unclear and represents a key area for future investigation. Second, while the single-cell translatome data confirmed translational asymmetry for RBBP7, this approach has not yet been extended to other candidate genes, leaving broader mechanistic insights unexplored. Third, the single-blastomere microinjection strategy does not control for pre-existing endogenous RBBP7 asymmetry between sister blastomeres. While this limitation is inherent to the method, the strong overexpression level and the reproducible results support the interpretability of the data. Future technical refinements, such as live imaging of endogenous protein prior to injection, could address this issue.

## Materials and methods

### Mouse oocytes and embryos collection

Embryos were collected from 4- to 6-week-old ICR mouse females mated with ICR males. To induce ovulation, females were administered 5 IU of human chorionic gonadotropin (hCG) intraperitoneally, 44−48 h after injection of 5 IU of pregnant mares’ serum gonadotropin (PMSG). Embryos were collected from female mice at the following time points post hCG injection: zygote stage (post hCG 19 hours), 2CE stage (post hCG 31−32 h), 2CM stage (post hCG 39−40 h), 2CL stage (E1.75, post hCG 46−48 h), 4-cell stage (E2.0, post hCG 54−56 h), 8 cell stage (E 2.5, post hCG 68−72 h), morula stage (E3.0, post hCG 84 h), and blastocyst stage (E3.5, post 96 h). Additionally, GV oocytes came from 6-week-old ICR females at post PMSG 44−48 h. MII oocytes were collected from 6-week-old ICR superovulated females at post hCG 14 h. To collect the single cells of embryos, the zona pellucida was removed using acidic Tyrode’s Solution (Sigma, T1788), embryos were washed twice using M2 medium (Millipore, MR-015-D), and then were continuously separated with 0.25% Trypsin-EDTA for several seconds. All animal experiments were approved by the Animal Care and Use Committee of China Agricultural University.

### Cell culture

Mouse ESCs (129/SvEv) were cultured on gelatin-coated plates in Dulbecco’s modified Eagle’s medium (DMEM) supplemented with 20% FBS (Gibco, 10099141 C), 2 mM GlutaMAX (Gibco, 35050061), 1× MEM non-essential amino acids (Gibco, 11140050), 1 mM sodium pyruvate (Gibco, 11360070), 100 μM β-mercaptoethanol (Gibco, 21985023), 1000 U/mL mLIF (Millipore, ESG1107), 3 μM CHIR99021 (Stemgent, 04-0004) and 1 μM PD0325901 (Stemgent, 04-0006).

### Microinjection

Before microinjection, the embryos were collected and incubated at Potassium Simplex Optimized Medium (KSOM) for 1 h in an incubator at 37 °C. About 1−2 pL siRNA (GenePharma) at 20 μM concentration or ASO (GenePharma) at 1 μM concentration was microinjected into the nucleus of zygote to knockdown *Rbbp7* or/and *LincGET*, using an eppendorf micromanipulator on a Nikon inverted microscope. About 1−2 pL mRNA with a 200 ng/μL concentration was microinjected into one of blastomeres in late 2-cell embryos to overexpress *LincGET*/*Rbbp7* or/and *Gfp*. The embryos were then observed and sampled at different time points. The sequences of *Rbbp7* siRNA (GenePharma) and *LincGET*-ASO were listed in Supplementary Table S8.

### Immunofluorescence staining (IF) and co-IF

After removal of the zona pellucida with acidic Tyrode’s Solution (Sigma, T1788), mouse embryos were fixed in 4% paraformaldehyde (PFA) buffer for 20 min and permeabilized with 0.25% Triton X-100 for 15 min at RT. The embryos were then blocked with blocking buffer (1% BSA in PBS) for 30 min at RT, and incubated with primary antibody diluted in blocking buffer for 2 h at 37 °C, followed by 3 washes with washing buffer (0.1% Tween-20 in PBS). Next, the embryos were incubated with Alexa Fluor-conjugated secondary antibody diluted in washing buffer for 1 h at 37 °C in the dark, followed by 3 washes. The nuclei were then stained with DAPI (10 mg/mL in PBS) for 5 min at RT, followed by 3 washes. Finally, the embryos were placed on the slides with one drop of mounting medium (Vector Laboratories, H-1200) and covered with coverslips.

Specially, for 3P-immunofluorescence, the embryos should first be incubated with 3P-M2 medium for appropriate time at 37 °C. And for puromycin-immunofluorescence, the embryos should first be incubated with 3 μM puromycin for 30 min at 37 °C.

For co-IF, most steps were the same as above, except that the antibody incubation required primary antibodies from different hosts and the corresponding secondary antibodies.

### Proximity ligation assay (PLA) and Puro-PLA

Duolink PLA Probes (Sigma, DUO92002 & DUO92004) and Duolink Fluorescent Detection Reagent (Sigma, DUO92008) were used for proximity ligation assay. The fixation, permeabilization, blocking and primary antibody incubation steps were consistent with immunofluorescence staining. Embryos were incubated in PLA probes diluent for 1 h at 37 °C. The ligation buffer and the amplification buffer were prepared according to the manufacturer’s instructions. After 3 washes, the embryos were incubated in the ligation buffer for 30 min at 37 °C, followed by amplification for 2 h at 37 °C protected from light. After final washes and DAPI staining, the embryos were mounted on glass slides for imaging.

Puro-PLA was performed according to the protocol of Dieck’s method^28^. In brief, embryos were first treated with 3 μM puromycin for 30 min at 37 °C, and the subsequent PLA procedure was performed in which anti-puromycin (Sigma, MABE343) and anti-RBBP7 (Abcam, ab259957) primary antibodies were used.

### RNA fluorescence in situ hybridization (RNA FISH) and IF-coFISH

For RNA FISH of *Rbbp7*, the following method was used. After removal of the zona pellucida with acidic Tyrode’s Solution (Sigma, T1788), embryos were incubated in PBS containing 6 mg/mL BSA for 5 min. Then the embryos were transferred onto coverslips using the mouth pipette and dried quickly^50^. Embryos were fixed in 4% PFA buffer for 15 min at RT, followed by permeabilization in permeabilizing buffer (0.2% Triton X-100, 10 mM VRC (Beyotime, R0108), in PBS) for 15 min at RT. After incubation in pre-hybridization buffer (40% Formamide (Sigma, F9037), 2× SSC (Promega, V4261), 10 mM VRC, 1 mg/mL BSA) for 30 min at 37 °C, the embryos were hybridized in prewarmed hybridization buffer (40% Formamide, 2× SSC, 10% Dextran sulfate (Sigma, D8906), 10 mM VRC, 1 mg/mL BSA) containing 100 nM biotin labeled probes (BGI) at 37 °C overnight. The probes were designed using PaintSHOP^51^ and listed in Supplementary Table S8. After hybridization, the embryos were washed three times with hybridization washing buffer (40% Formamide, 2× SSC) for 5 min each at 42 °C, followed by 3 washes with 2× SSC for 5 min each at RT. Next, the embryos were blocked in blocking buffer (1% BSA in PBS) for 15 min at 37 °C, and incubated with streptavidin-Cy3 (Sigma, S6402) diluted in blocking buffer (1:100) for 30 min at 37 °C. After 3 washes with washing buffer (0.1% Tween-20 in PBS), the embryos were stained with DAPI, and the coverslips were finally mounted for imaging.

For RNA FISH and IF-coFISH of *LincGET*, we performed the assays according to a previously reported method^12^. Briefly, probes were synthetized by in vitro transcription using MEGAshortscript Kit (Ambion, AM1354), in which 25% of the UTPs were replaced with ChromaTide Alexa Fluor 488-5-UTPs (Invitrogen, C11403). The treatment of the embryos before fixation was the same as RNA FISH of *Rbbp7*. The embryos were fixed and permeabilized with 1% PFA and 0.04% NP-40 on ice for 5 min, followed by fixation with 1% PFA for 5 min. If immunofluorescence staining was needed, an additional fixation in 4% PFA for 10 min should be performed after washes of the secondary antibody, and RNase inhibitor should be added into the buffers in every step. The dehydration of the embryos was carried out in 70%, 80%, 95% and 100% Ethanol, with each incubation lasting for 5 min at RT. Next, the embryos were hybridized in hybridization solution (50% Formamide, 2× SSC, 10% Dextran Sulfate, 10 mM VRC, 1 mg/mL BSA) containing 0.5 ng/µL fluorescence probes at 37 °C overnight. After the hybridization, the embryos were washed three times with hybridization washing solution (50% Formamide, 2× SSC) for 5 min at 45 °C each, followed by 3 washes with 2× SSC for 5 min each. Finally, the embryos were stained with DAPI, and mounted on glass slides after 3 washes.

### Microscopic analysis and three-dimensional image processing

Images were acquired using either a Leica TCS SP8 confocal microscope or a Nikon A1 confocal microscope. The relative mean fluorescence densities of proteins were analyzed by ImageJ and plotted using GraphPad Prism software (v8.0). For the quantification of the number of PLA signals through the entire embryos, three-dimensional confocal images were taken and maximum intensity projection method was applied using ImageJ. Imaris Viewer software (v.9.9.0) was used to capture the three-dimensional view of morula or blastocyst.

### RT-qPCR

Total RNA of cells or embryos was extracted by Trizol reagent (Invitrogen, 15596018) with accordance to the standard protocol. The pure RNA was dissolved with nucleotide free water for the 1^st^ cDNA synthesis using First Strand cDNA Synthesis Kit (Thermo, K16225). Ultimately, the cDNA was amplified with ChamQ Universal SYBR qPCR Master Mix (Vazyme, Q711-02). Samples were quantified by an AriaMx Real-Time PCR System (Agilent Technologies). *18S* rRNA was used as an internal control. The sequences of primers used in this study were listed in Supplementary Table S8.

### Western blotting

Total proteins were extracted using RIPA lysis buffer (Beyotime, P0013B) supplemented with protease inhibitor cocktail (Bimake, B14002), separated on SDS-PAGE and transferred to PVDF membranes (Millipore, ISEQ10100). After blocking with 5% non-fat dried milk in TBST and incubating with the primary and secondary antibodies diluted in blocking buffer, the membranes were visualized by ECL Prime Western blotting detection reagent (Cytiva, RPN2232). Quantification of each band was carried out using ImageJ.

### Immunoprecipitation coupled to mass spectrometry (IP-MS)

Approximately 50 million mESCs were used for immunoprecipitation of endogenous RBBP7 and IgG control. The cell pellet was resuspended in 500 μL cell lysis buffer (Beyotime, P0013) supplemented with protease inhibitor cocktail (Bimake, B14002) and gently rotated for 30 min at 4 °C. After centrifugation, the supernatant was collected into a new tube. To preclear the cell lysate, 10 μL Protein A/G beads (Pierce, 88803) were used, with gentle rotation for 30 min at 4 °C. Next, the pre-cleared cell lysate was divided into two aliquots, either 2 μg anti-RBBP7 antibody (Abcam, ab259957) or 2 μg normal rabbit IgG (Millipore, 12-370) was used for reaction with gentle rotation at 4 °C overnight. Then each sample was mixed with 10 μL pre-blocked Protein A/G beads and gently rotated for 1 h at room temperature (RT). The collected beads were washed three times with washing buffer (50 mM Tris-HCl pH 7.4, 150 mM NaCl, 1% NP-40, 1% glycerol), followed by 2 washes with high-salt washing buffer (50 mM Tris-HCl pH 7.4, 500 mM NaCl, 1% NP-40, 1% glycerol) and 1 wash with purified water. The samples were eluted with SDS-PAGE reducing loading buffer (CWBIO, CW0027) for 10 min at 95 °C. Finally, the samples were subjected to 10% SDS-PAGE gel and visualized by Coomassie blue staining. The protein-containing gel slices were applied to mass spectrometry analysis (Institute of Biophysics, CAS) using a nanoLC-Orbitrap Exploris 480 (Thermo Scientific).

### Single-cell cleavage under targets and tagmentation

scCUT&Tag was performed in accordance with the protocol of NovoNGS CUT&Tag 3.0 High-Sensitivity kit (Novoprotein, N259-YH01). Briefly, the mouse embryos were digested to individual cells as previously described, and single cells were then picked into single tubes, incubated with well-washed ConA beads for 10 min at RT. The cell-beads were then incubated with primary antibody on a rotator for 2 h at RT or overnight at 4 °C, followed by incubation with secondary antibody on a rotator for 1 h at RT. The ChiTag pAG-Tn5 was added to resuspend the cell-beads after twice washes, with incubation on rotator for 1 h at RT. The tagmentation was then performed by adding the tagmentation buffer following twice washes with ChiTag buffer. The fragmented DNA was extracted by DNA extract beads, and PCR was performed using i5 and i7 primers, followed by the library purification with DNA Clean beads (Vazyme, N411-01) and validation using the Agilent 2100 bioanalyzer.

### Chemical synthesis of 3P

The synthesis of 3P molecule was followed previously reported procedures^24^ with the following modifications. Briefly, NHS was added to a solution of Biotin and DCC in DMF/pyridine, stirred to obtain a white precipitate (B-NHS). One amino group of jeffamine was protected by BOC (J-BOC) through the reaction with (BOC)_2_O. Then, B-NHS, J-BOC and TEA were dissolved in dry DMF to yield a canary yellow solid (BJ1-BOC). The compound (BJ1-BOC) was dissolved in DCM, and BOC deprotection was performed using TFA, to obtain the product BJ1. Meanwhile, J-BOC was dissolved in dry acetonitrile and reacted with CDI to obtain a product called J-BOC’. The acetonitrile solution of J-BOC’ was directly added into the reaction flask of BJ1, together with DIPEA, to yield a canary yellow solid (BJ2-BOC). Through BOC deprotection and activation with CDI, BJ2-BOC was finally connected to puromycin. The product was purified by flash column chromatography to give 3P as a white foam solid.

### Single-cell puromycin-based ribosome sequencing

The single cells were incubated in medium with 3P (500 μM) at 37 °C for 15-30 min, and washed twice with M2 medium. Then, the 3P-labeled single cells were transferred into a low-adsorption 1.5-mL tube containing 20 μL lysis buffer (20 mM Tris-HCl, pH 7.4, 150 mM NaCl, 5 mM MgCl_2_, 1 mM DTT, 1% Triton X-100) supplemented with 5 U/mL DNase (Ambion, AM2239), protease inhibitor cocktail (Bimake, B14002) and 200 U/mL RNase inhibitor (Beyotime, R0102-50kU) by a mouth pipette, and incubated for 30 min on ice. To roughly digest the mRNA unprotected by ribosomes, 0.6 μL RNase I (Ambion, AM2295) was added to each tube and reacted for 30 min at RT with gentle rotation. The reaction was stopped by adding 0.6 μL SUPERase•In (Ambion, AM2694). Then, 2 μL pre-washed streptavidin-beads (Invitrogen, 65002) was added into the tube and incubated for 1 h or overnight at 4°C with gentle rotation. The tube was then put on a magnetic stand for 5 min to collect the beads-ribosomes and washed four times with washing buffer (150 mM NaCl, 10 mM of Tris-HCl, pH 7.5, 1% Triton X-100) supplemented with protease inhibitor cocktail and 200 U/mL RNase inhibitor. The beads-ribosomes were resuspended with 200 μL PK buffer (100 mM Tris-HCl, pH 7.5, 50 mM NaCl, 10 mM EDTA, 1% SDS) containing 2 mg/mL proteinase K (Roche, 3115828001), and incubated on the constant temperature oscillating metal bath for 1 h at 55 °C with 1100 rpm rotation. RNAs were then extracted by acidic-phenol chloroform separation. The library construction was performed according to the manufacturer’s instructions of SMARTer® Stranded Total RNA-seq Kit v2 (Takara, 634414).

### Single-cell RNA sequencing

Total RNA of single cell was extracted using Trizol reagent (Invitrogen, 15596018), purified by alcohol precipitation, and directly subjected to library construction according to the manufacturer’s instructions of SMARTer® Stranded Total RNA-seq Kit v2 (Takara, 634414) with fragmentation. Sequencing was performed on an Illumina NovaSeq6000 platform.

### High throughput sequencing data processing

scpRibo-seq, scRNA-seq, and scCUT&Tag were carried out on Illumina NovaSeq6000 platform with paired-end 150 bp read length. Cutadapt (v.1.13, default parameters) (https://cutadapt.readthedocs.io/en/stable/)^52^ was used to trim off adapter sequences, and reads < 25 nt in length were filtered out by Trimmomatic (v.0.33, parameters: LEADING:20, TRAILING:20 SLIDINGWINDOW:4:15, MINLEN:25, HEADCROP:3)^53^. Clean reads of scpRibo-seq and scRNA-seq were first mapped to mouse genome (mm10, Ensembl) using Bowtie 2 (v.2.2.9, default parameters) or TopHat (v.2.1.1, default parameters)^54,55^, respectively. Reads that aligned to rRNAs, tRNAs, snRNAs, and snoRNAs were discarded. Sequences for rRNAs and tRNAs were downloaded from NCBI RefSeq and tRNAdb (http://trna.bioinf.uni-leipzig.de/DataOutput/)^56^, respectively. The number of reads mapped to each gene was counted using the HTSeq (v.0.11.2) with parameters: -m union -s no. RPKM was computed as the number of reads which map per kilobase of exon model per million mapped reads for each gene. The Ribo-seq (GSE102318)^57^, RiboLace (GSE102354)^24^, and LiRibo-seq (GSE169632)^25^ data were downloaded from the GEO database.

Clean reads of scCUT&Tag were mapped to mouse genome (mm10, Ensembl) using Bowtie2 (v.2.2.9, default parameters)^54^. MACS2 (v.2.1.4)^58,59^ was used for the peak calling with the parameters: -c -f BAM --nomodel -gsize = mm --keep-dup all -n -p 1e-5 -B. analyzeRepeats.pl. HOMER (v.4.9.1, default parameters)^60^ was performed to do peak annotation and calculate the raw read counts (RPM) of gene bodies and promoters (defined as the regions 3 kb from the TSS). The metagene profile was generated using ngsplot (v.2.61) with parameters: -G mm10 -R gene body -L 3000^61^. The signal intensity of each 2 kb window was calculated as the value reads per million bases (RPM). Genes with fold change of H3K9ac signals between two sister cells in each embryo over 1.5 and *P* < 0.05 (Student’s *t*-test and Wilcoxon and Mann-Whitney test) were identified as differential H3K9ac-modified genes.

## Supplementary information

Supplementary information, Figures and Tables

Supplementary Table S3

Supplementary Table S5

## Data Availability

High-throughput sequencing (scRNA-seq, scpRibo-seq and scCUT&Tag) data that support the findings of this study have been deposited at Genome Sequence Archive of National Genomics Data Center^62^ under the accession number CRA007727 linked to the project PRJCA010768. All expression plasmids used in this study will be made available upon request. This study did not generate new, unique reagents. This study did not generate new code.
