# Supplementary material for: Asymmetric RBBP7 regulates the first cell fate decision of early mammalian embryos
Source: Cell Discov. 2026 Aug 18;12:60. doi: 10.1038/s41421-026-00912-6 (PMC13482399; doi:10.1038/s41421-026-00912-6)
Supplement: Supplementary file 1 [file 41421_2026_912_MOESM1_ESM.pdf]

Supplementary Information

Asymmetric RBBP7 regulates the first cell fate decision of early mammalian embryos

Author list

Lin-Fang Ju, Xiao Han , Danru Zhang, Heng-Ji Xu, Shaokang Jia, Meng-Xia Liu, Yanchen, Liu, Yu-Ting Cai, Yu-Sheng Chen, Chun-Chun Gao, Yong-Liang Zhao, Yajing Hao, Xiu-Jie Wang, Dangsheng Li, Xiang Zhou, Ying Yang, Jianyong Han & Yun-Gui Yang

This PDF includes：

Supplementary Figures S1 to S7 and Supplementary Figure legends

Supplementary Tables

Table S1, S2, S4, S6, S7, S8

Supplementary Table legends

Table S3 and S5

Supplementary Figures and Figure legends

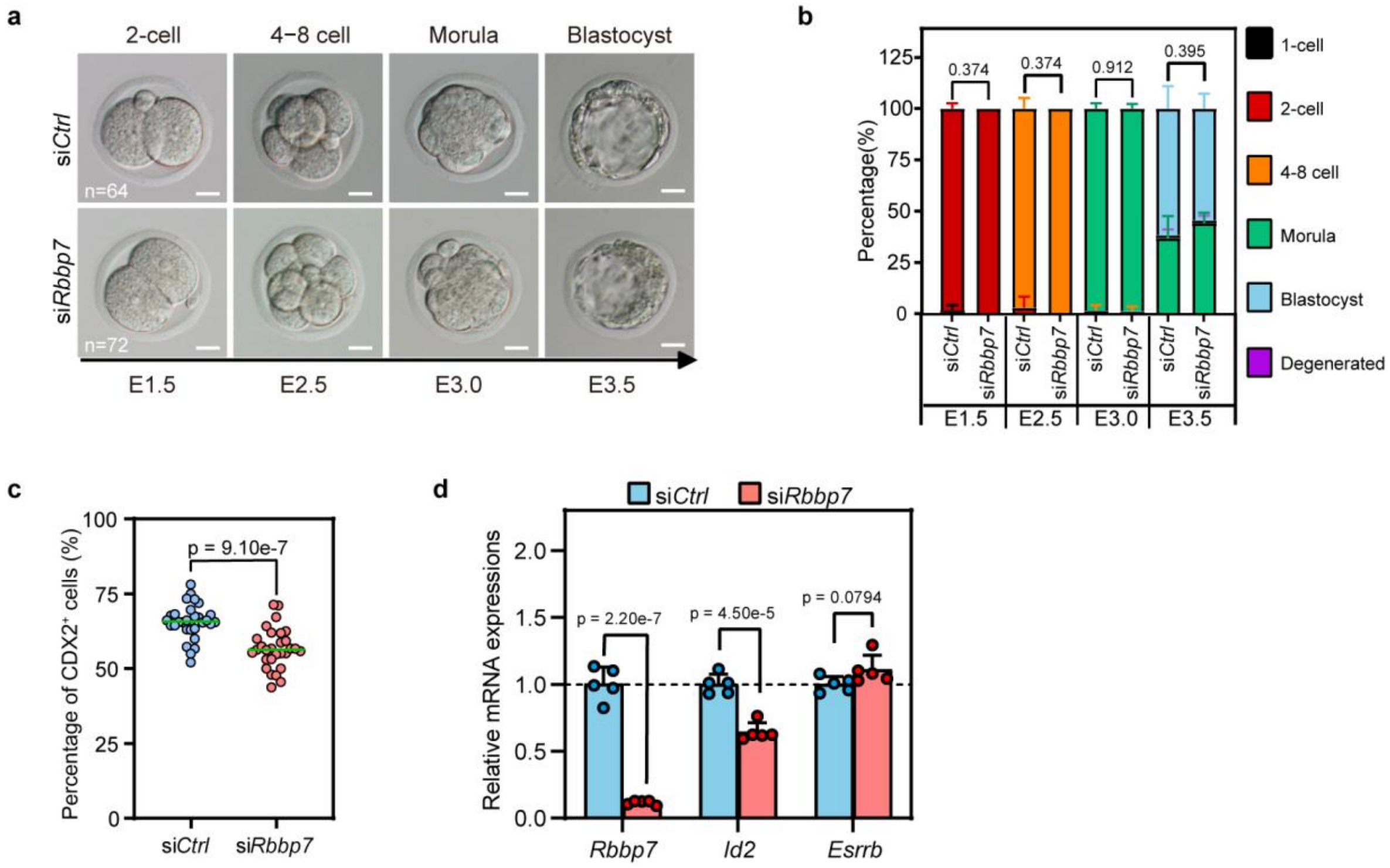

Fig. S1. *Rbbp7* is closely related to embryonic cell fate. a The stereomicroscope images of the control embryos and *Rbbp7* deficient embryos at different mouse developmental stages. Scale bar, 20 µm. Scale bar, 20 µm. **b** Statistic analysis of (**c**) showing the ratios of embryos at different mouse developmental timepoints. (Three replicate; control embryos: n = 64, *Rbbp7* deficient embryos: n = 72; two-sided unpaired Student's *t* test). Error bar represents SEM. **c** Quantification of percentage of $CDX2^+$ cells by 3D reconstruction in Figure 1f. The p values were determined by the Student's *t* test. $CDX2^+$ cells were defined as those with higher fluorescence for CDX2 than SOX2. Double-positive ($SOX2^+$ $CDX2^+$) cells were excluded from this analysis. **d** Barplots showing the mRNA fold changes of *Rbbp7*, *Id2*, and *Esrrb* detected by RT-qPCR in si*Ctrl* and si*Rbbp7* embryos. The fold change of each gene was normalized to the expression level of 1*8S* rRNA. The p values were determined by the Student's *t* test.

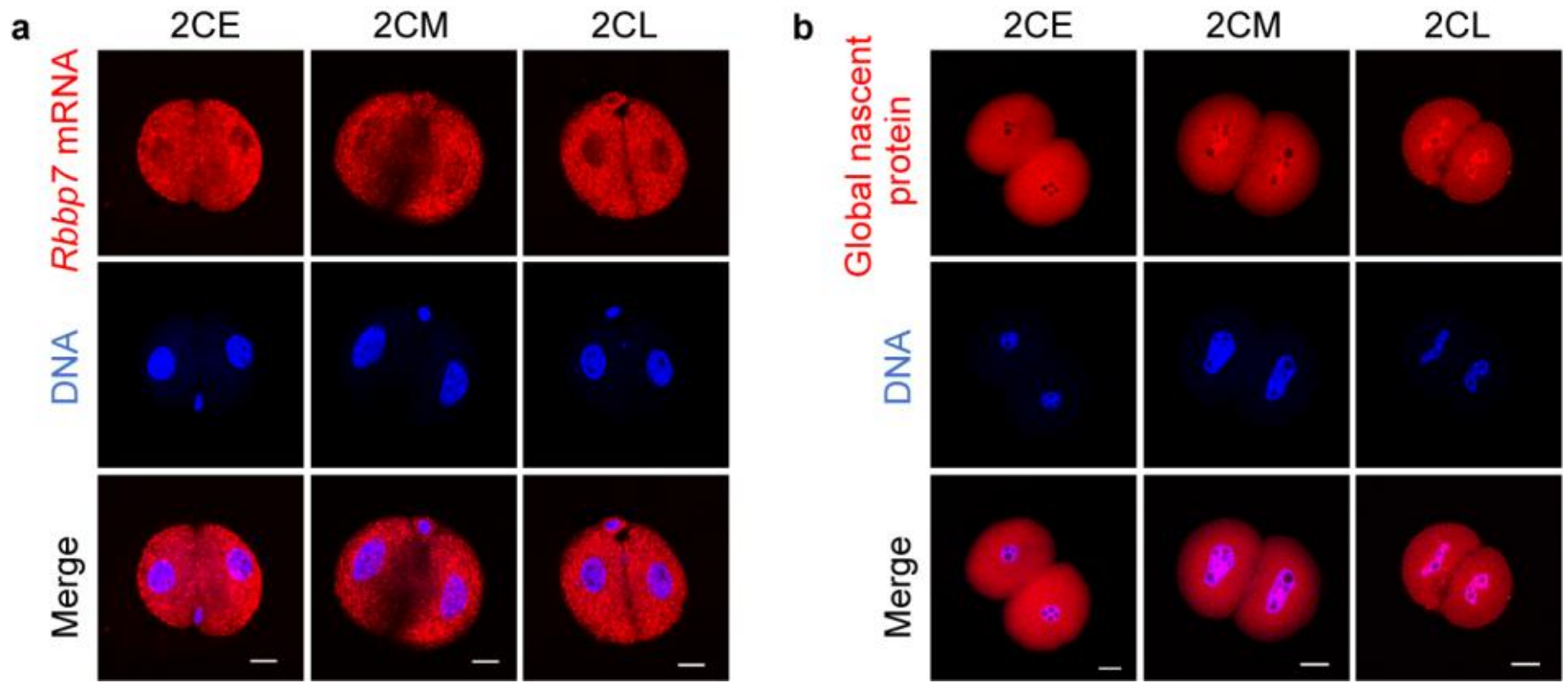

Fig. S2. Heterogenous RBBP7 distribution is established by asymmetric *Rbbp7* translation, not transcriptional heterogeneity. a RNA-FISH staining of *Rbbp7* showing the RNA expression levels of *Rbbp7* in 2CE, 2CM, and 2CL embryos. Scale bar, 20 μm. **b** Puromycin immunofluorescence staining showing the global nascent protein synthesis in 2CE, 2CM, and 2CL embryos.

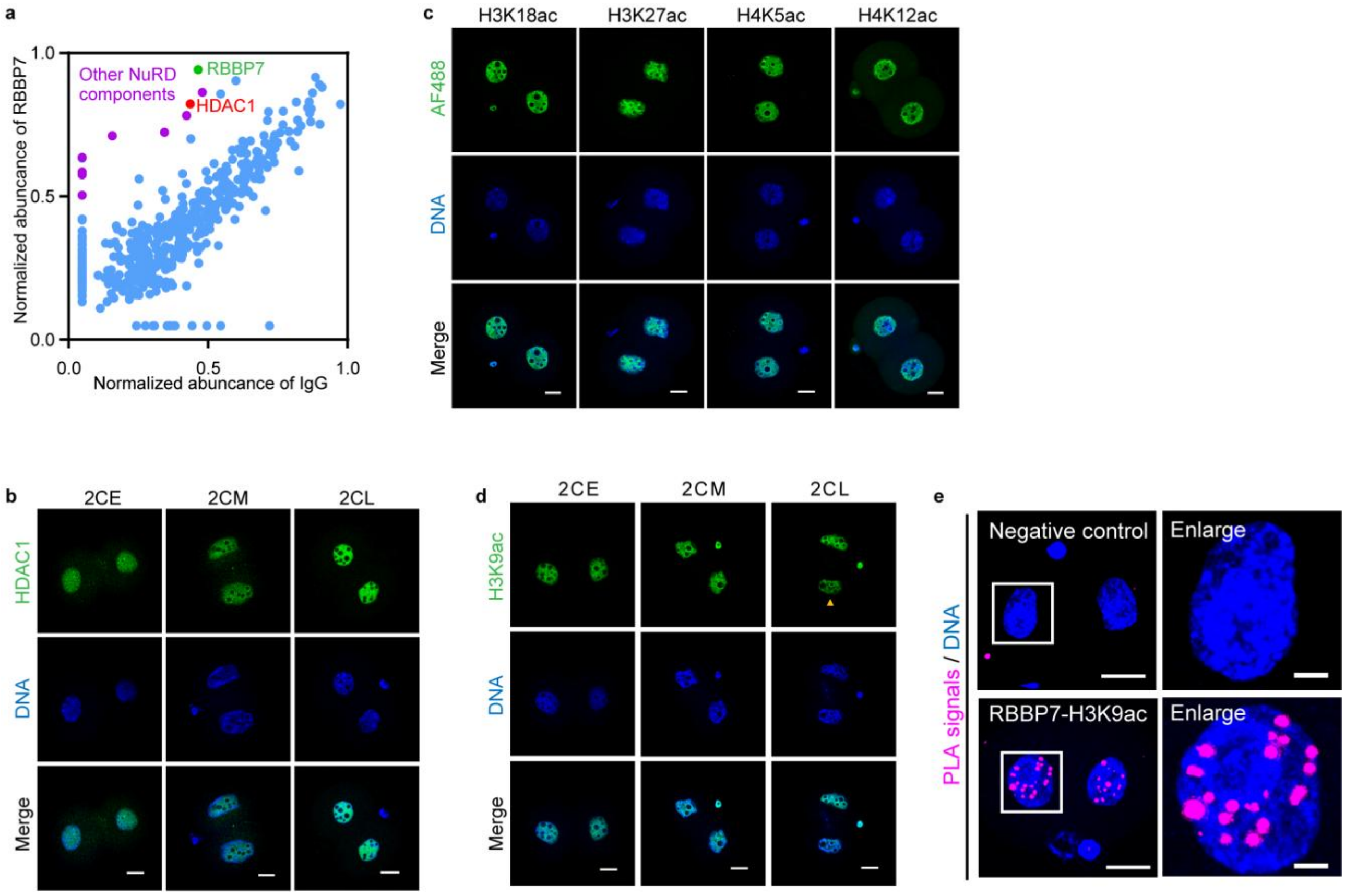

Fig. S3. Asymmetry of RBBP7, not HDAC1, leads to the opposite H3K9ac level in 2-cell embryos. **a** IP-MS analysis of the interactional proteins of RBBP7. Rabbit IgG was used as control, and anti-RBBP7 antibody was used to enrich RBBP7 targets. Great enrichment of HDAC1(red) and other NuRD complex components (purple) was observed in RBBP7 antibody group compared to IgG control group. **b** Immunofluorescence staining of H3K18ac, H3K27ac, H4K5ac, and H4K12ac in 2CL embryos. Scale bar, 20 μm. **c** Immunofluorescence staining of HDAC1 in 2CE, 2CM, and 2CL embryos. Scale bar, 20 μm. **d** Immunofluorescence staining of H3K9ac in 2CE, 2CM, and 2CL embryos. The yellow triangle indicates blastomere with relatively less H3K9ac. Scale bar, 20 μm. **e** Confocal images of RBBP7-H3K9ac PLA in 2CL embryos. Scale bar, 20 μm (left); 5 μm (right, enlarged region).

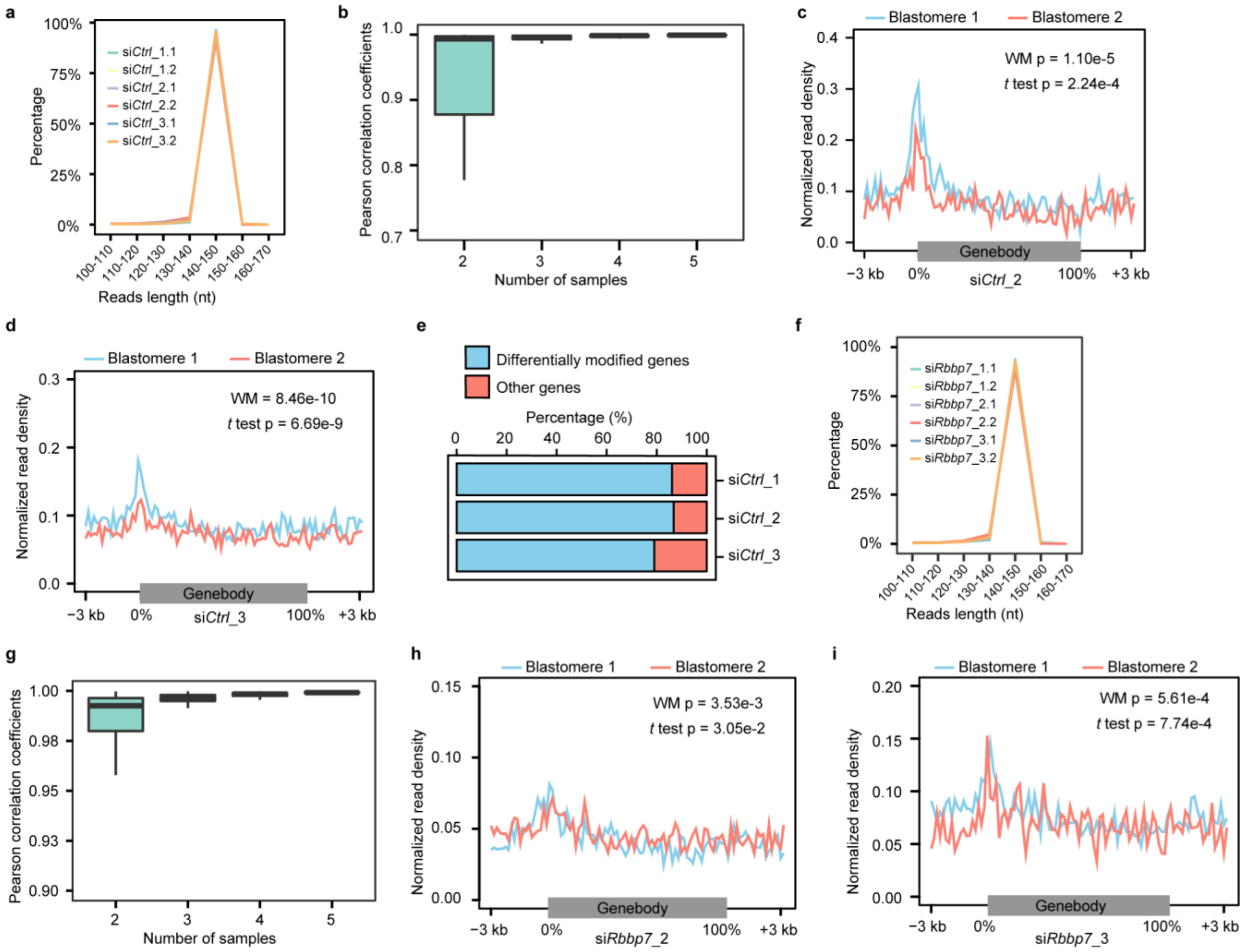

Fig. S4. *Rbbp7* knockdown abolishes cellular heterogeneity of H3K9ac. a Percentage of different lengths of mapped reads in si*Ctrl* samples from scCUT&Tag data. **b** Boxplot displaying the correlation of H3K9ac signals between single cell samples and randomly extracted single cell samples in si*Ctrl* embryos. **c, d** Metagene profiles of normalized H3K9ac read density in two blastomeres of si*Ctrl*_2 (**c**) and si*Ctrl*_3 (**d**) embryos (WM refers to two-sided Wilcoxon and Mann-Whitney test, *t* test refers to two-sided paired Student's *t* test). **e** Percentage of differential H3K9ac modified genes between blastomeres from si*Ctrl* embryos. **f** Percentage of different lengths of mapped reads in si*Rbbp7* samples from scCUT&Tag data. **g** Boxplot displaying the correlation of H3K9ac signals between single cell samples and randomly extracted single cell samples in si*Rbbp7* embryos. **h, i** Metagene profiles of normalized H3K9ac read density in two blastomeres of si*Rbbp7*_2 (**j**) and si*Rbbp7*_3 (**k**) embryos (WM refers to two-sided Wilcoxon and Mann-Whitney test, *t* test refers to two-sided paired Student's *t* test).

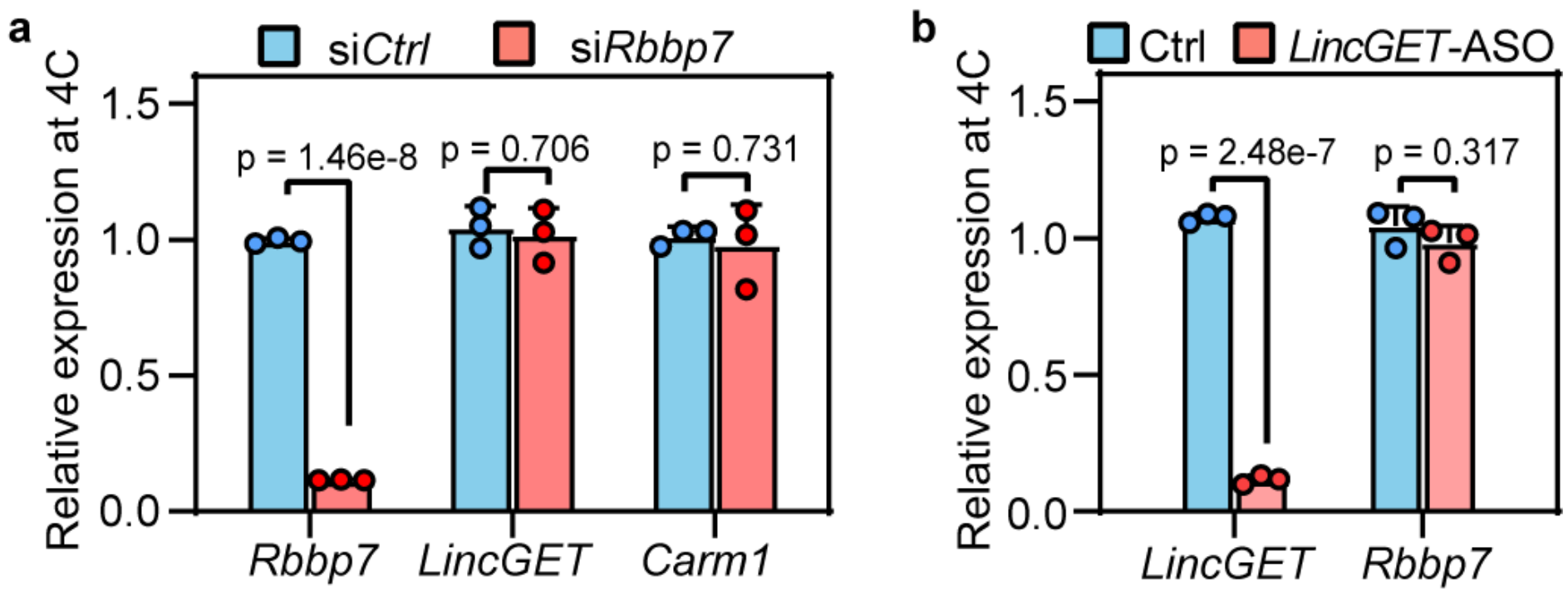

Fig. S5. RBBP7 and *LincGET* collaboratively regulate early cell fate bias. a Barplots of RNA fold changes of *Rbbp7*, *LincGET*, and *Carm1* determined by RT-qPCR (n = 60 for three replicates; Student's *t* test). Normalized to the expression level of *18S* rRNA. Error bar represents SEM. **b** Barplots of RNA fold changes of *LincGET* and *Rbbp7* determined by RT-qPCR (n = 60 for three replicates; Student's *t* test). Normalized to the expression level of *18S* rRNA. Error bar represents SEM.

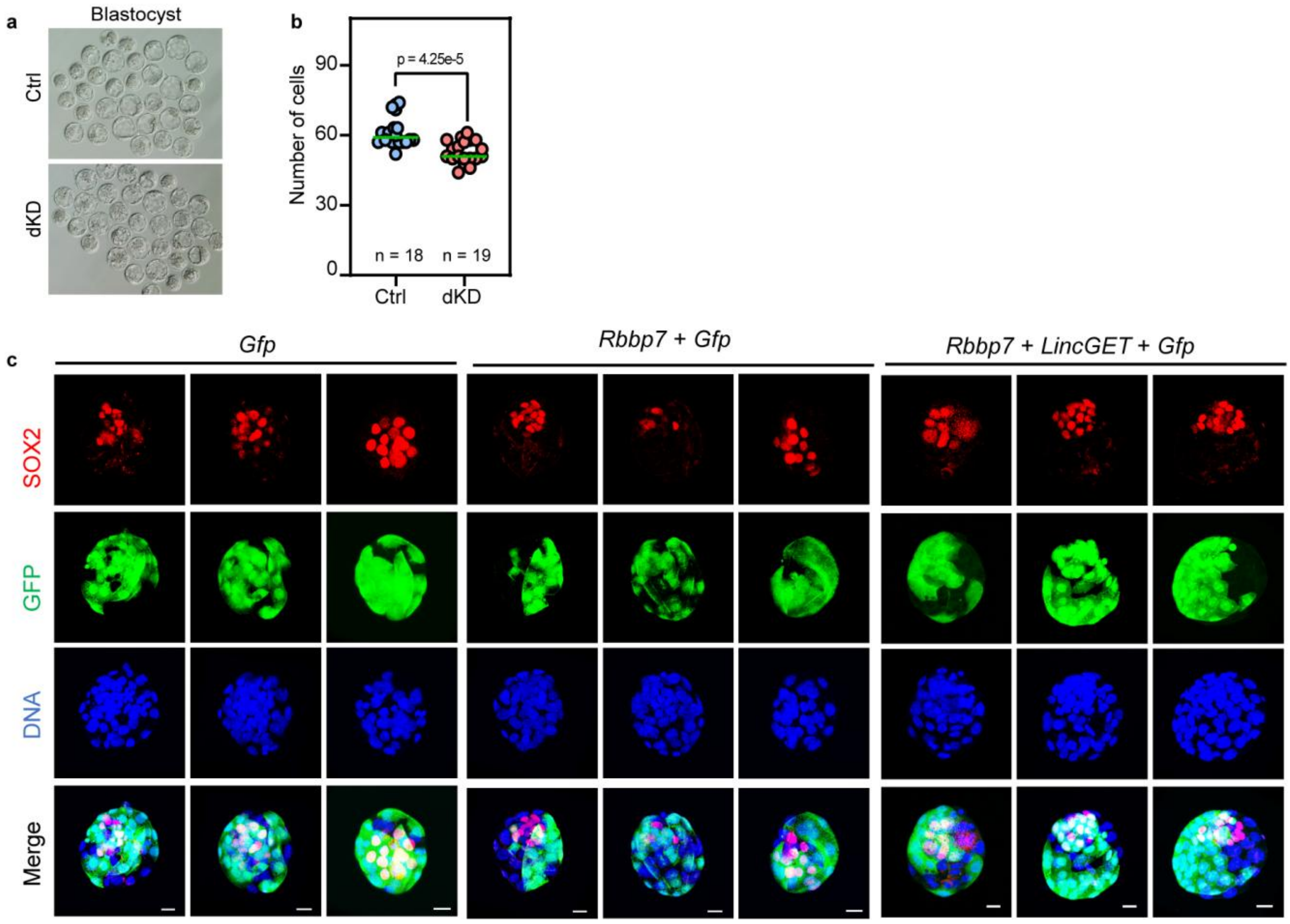

Fig. S6. *Rbbp7* and *LincGET* collaboratively regulate early cell fate decision. a The stereomicroscope images of control embryos and *Rbbp7*-*LincGET* deficient embryos at mouse blastocyst stage. Scale bar, 20 µm. **b** The quantification analysis of the cell numbers in **Fig. 7b** (Student's *t* test). **c** The fluorescent staining of SOX2 and GFP in blastocysts. SOX2 served as a marker for ICM cells. Scale bar, 20 µm.

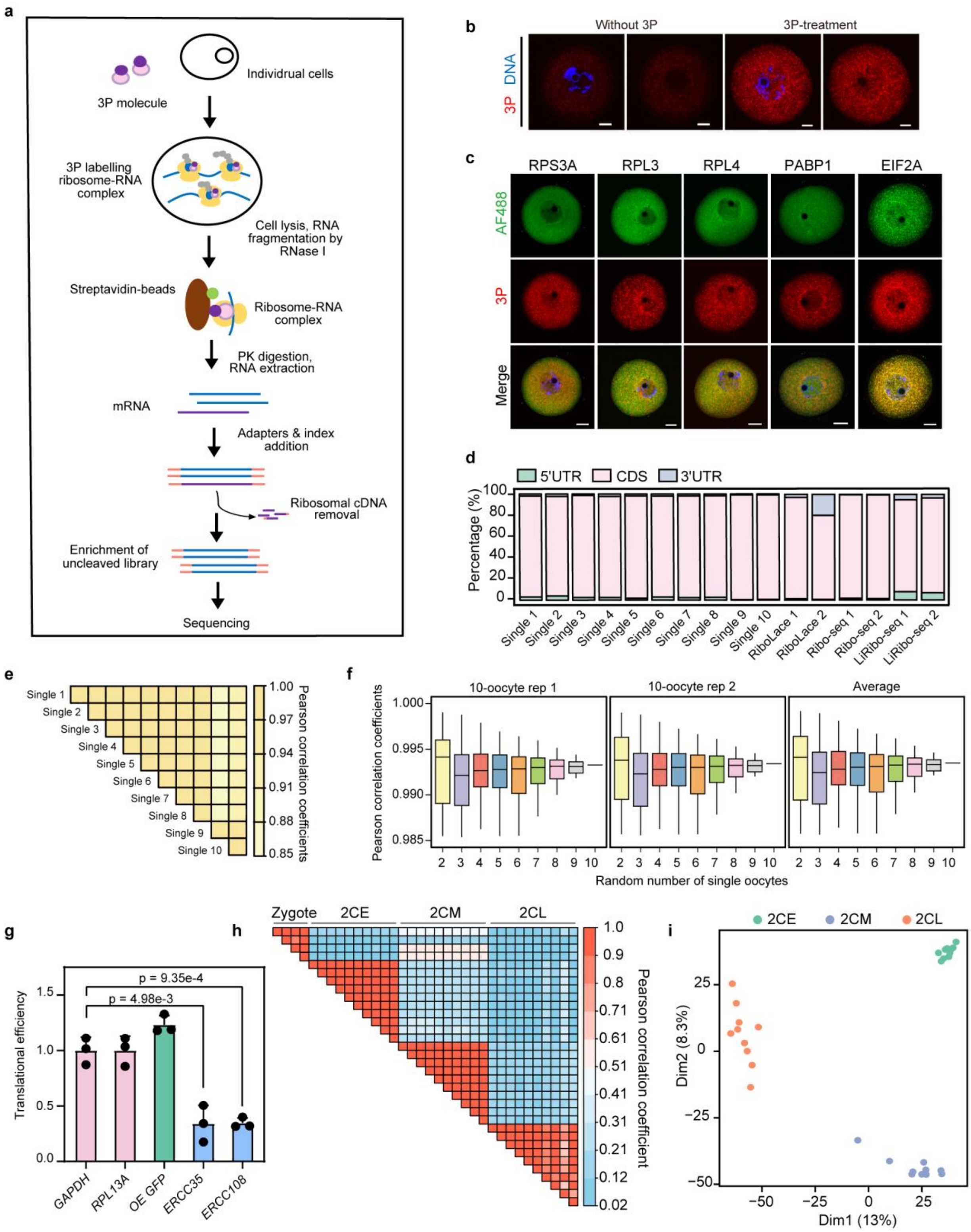

Fig. S7. scpRibo-seq profiles translation in single oocyte and single cells of 2-cell embryos. a Schematic of the scpRibo-seq workflow. **b** Immunofluorescence staining showing the incorporation of 3P into GV oocytes. The oocytes were incubated with 3P molecules. Scale bar, 20 μm. **c** Co-immunofluorescence staining of translation related proteins and 3P molecule. Scale bar, 20 μm. **d** Percentage of aligned reads in scpRibo-seq, Ribo-seq, RiboLace, and LiRibo-seq along the 5'UTR, CDS, and 3'UTR. Mb represents mouse brain sample. **e** Heatmap showing the correlation of

replicates from scpRibo-seq data of 10 single oocytes. **f** Boxplots displaying the correlation of translation signals of randomly extracted single cell samples (number of extracted oocytes = 2, 3, 4, …, 10) with 10-oocyte replicate 1 (left), replicate 2 (middle), and their average (right). **g** Barplots showing the relative translation efficiency (translation/transcription) of translating genes, overexpressed (OE) GFP, and the incorporated ERCC RNAs. **h** Heatmap showing the correlation of different replicates at zygote, 2CE, 2CM, and 2CL stages from scpRibo-seq. **i** Principal component analysis (PCA) with cells at 2CE, 2CM, and 2CL stages using scpRibo-seq data.

Supplementary Table legends

Supplementary Table S1. Analysis of number of cells for 3D images of si*Ctrl* and si*Rbbp7* embryos, related to Fig. 1.

E3.0				E3.5			
Sample	Total cells	Sample	Total cells	Sample	Total cells	Sample	Total cells
si*Ctrl*-1	18	si*Rbbp7*-1	32	si*Ctrl*-1	62	si*Rbbp7*-1	47
si*Ctrl*-2	31	si*Rbbp7*-2	31	si*Ctrl*-2	62	si*Rbbp7*-2	41
si*Ctrl*-3	33	si*Rbbp7*-3	28	si*Ctrl*-3	64	si*Rbbp7*-3	52
si*Ctrl*-4	30	si*Rbbp7*-4	17	si*Ctrl*-4	66	si*Rbbp7*-4	43
si*Ctrl*-5	33	si*Rbbp7*-5	31	si*Ctrl*-5	65	si*Rbbp7*-5	54
si*Ctrl*-6	25	si*Rbbp7*-6	29	si*Ctrl*-6	66	si*Rbbp7*-6	59
si*Ctrl*-7	18	si*Rbbp7*-7	22	si*Ctrl*-7	65	si*Rbbp7*-7	49
si*Ctrl*-8	28	si*Rbbp7*-8	26	si*Ctrl*-8	67	si*Rbbp7*-8	61
si*Ctrl*-9	24	si*Rbbp7*-9	18	si*Ctrl*-9	62	si*Rbbp7*-9	52
si*Ctrl*-10	25	si*Rbbp7*-10	32	si*Ctrl*-10	61	si*Rbbp7*-10	49
si*Ctrl*-11	26	si*Rbbp7*-11	33	si*Ctrl*-11	60	si*Rbbp7*-11	64
si*Ctrl*-12	30	si*Rbbp7*-12	17	si*Ctrl*-12	60	si*Rbbp7*-12	65
si*Ctrl*-13	33	si*Rbbp7*-13	29	si*Ctrl*-13	64	si*Rbbp7*-13	55
si*Ctrl*-14	32	si*Rbbp7*-14	30	si*Ctrl*-14	56	si*Rbbp7*-14	42
si*Ctrl*-15	31	si*Rbbp7*-15	26	si*Ctrl*-15	61	si*Rbbp7*-15	61
si*Ctrl*-16	33	si*Rbbp7*-16	25	si*Ctrl*-16	65	si*Rbbp7*-16	57
si*Ctrl*-17	31	si*Rbbp7*-17	27	si*Ctrl*-17	73	si*Rbbp7*-17	62
si*Ctrl*-18	33	si*Rbbp7*-18	17	si*Ctrl*-18	72	si*Rbbp7*-18	65
si*Ctrl*-19	32			si*Ctrl*-19	73	si*Rbbp7*-19	60
si*Ctrl*-20	27					si*Rbbp7*-20	67
si*Ctrl*-21	32					si*Rbbp7*-21	55
si*Ctrl*-22	25					si*Rbbp7*-22	63
si*Ctrl*-23	32						
si*Ctrl*-24	32						

Supplementary Table S2. Analysis of SOX2 intensity and SOX2$^+$ cell number for 3D images of si*Ctrl* and si*Rbbp7* embryos, related to Fig. 1.

Sample	DNA$^+$	SOX2$^+$	CDX2$^+$	SOX2$^+$ (%)	SOX2 intensity	Sample	DNA$^+$	SOX2$^+$	CDX2$^+$	SOX2$^+$ (%)	SOX2 intensity
si*Ctrl*-1	57	18	36	31.58	2420.41	si*Rbbp7*-1	60	21	34	35.00	2223.55
si*Ctrl*-2	57	15	41	26.32	3782.59	si*Rbbp7*-2	56	21	35	37.50	2743.81
si*Ctrl*-3	62	15	42	24.19	1868.04	si*Rbbp7*-3	45	13	32	28.89	1219.23
si*Ctrl*-4	61	22	40	36.07	2343.98	si*Rbbp7*-4	43	21	24	48.84	2580.82
si*Ctrl*-5	61	29	35	47.54	1164.91	si*Rbbp7*-5	56	20	36	35.71	5306.60
si*Ctrl*-6	65	21	43	32.31	1260.89	si*Rbbp7*-6	44	18	22	40.91	6271.10
si*Ctrl*-7	53	16	35	30.19	1261.71	si*Rbbp7*-7	55	17	37	30.91	4045.52
si*Ctrl*-8	60	25	34	41.67	2781.68	si*Rbbp7*-8	50	21	24	42.00	1271.96
si*Ctrl*-9	54	20	34	37.04	696.57	si*Rbbp7*-9	49	20	27	40.82	4830.11
si*Ctrl*-10	55	12	43	21.82	3573.96	si*Rbbp7*-10	49	16	35	32.65	2611.40
si*Ctrl*-11	69	32	36	46.38	550.13	si*Rbbp7*-11	50	19	31	38.00	1366.40
si*Ctrl*-12	68	18	51	26.47	1042.06	si*Rbbp7*-12	63	25	37	39.68	2711.43
si*Ctrl*-13	75	30	45	40.00	2172.71	si*Rbbp7*-13	64	31	32	48.44	2010.59
si*Ctrl*-14	62	22	40	35.48	2422.39	si*Rbbp7*-14	60	24	36	40.00	6659.96
si*Ctrl*-15	60	21	38	35.00	2365.72	si*Rbbp7*-15	60	26	34	43.33	905.39
si*Ctrl*-16	58	20	38	34.48	2485.25	si*Rbbp7*-16	58	26	32	44.83	1913.42
si*Ctrl*-17	50	15	34	30.00	1184.47	si*Rbbp7*-17	42	16	26	38.10	2392.11
si*Ctrl*-18	64	17	47	26.56	1471.86	si*Rbbp7*-18	71	31	40	43.66	2906.02
si*Ctrl*-19	58	17	38	29.31	2390.72	si*Rbbp7*-19	47	16	27	34.04	2299.15
si*Ctrl*-20	64	21	43	32.81	3449.44	si*Rbbp7*-20	56	19	31	33.93	3023.81
si*Ctrl*-21	60	21	33	35.00	878.74	si*Rbbp7*-21	64	36	28	56.25	4931.04
si*Ctrl*-22	60	21	39	35.00	2772.32	si*Rbbp7*-22	60	26	34	43.33	5665.99
si*Ctrl*-23	71	19	52	26.76	557.00	si*Rbbp7*-23	62	28	34	45.16	2282.95
si*Ctrl*-24	83	27	56	32.53	1676.45	si*Rbbp7*-24	66	29	35	43.94	4540.02
si*Ctrl*-25	63	18	43	28.57	3044.38	si*Rbbp7*-25	54	22	32	40.74	4012.35
si*Ctrl*-26	59	24	38	40.68	859.46	si*Rbbp7*-26	47	22	25	46.81	5479.52
si*Ctrl*-27	62	21	41	33.87	1500.78	si*Rbbp7*-27	64	23	36	35.94	5122.56
si*Ctrl*-28	66	20	46	30.30	1228.69	si*Rbbp7*-28	44	19	21	43.18	2775.37
si*Ctrl*-29	84	26	55	30.95	936.49	si*Rbbp7*-29	57	18	26	31.58	1547.92
si*Ctrl*-30	62	21	41	33.87	2229.27						

Supplementary Table S4. Data information of scCUT&Tag samples, related to Fig. 4.

Sample	Total reads	HQ reads	HQ reads (%)	Mapping reads	Mapping reads (%)
si*Rbbp7*_1.1	42348179	41995089	99.17%	13346039	31.78%
si*Rbbp7*_1.2	51704316	51308085	99.23%	8558189	16.68%
si*Rbbp7*_2.1	47239633	46859474	99.20%	24179489	51.60%
si*Rbbp7*_2.2	44363547	43676044	98.45%	23095892	52.88%
si*Rbbp7*_3.1	47583605	47260031	99.32%	21994818	46.54%
si*Rbbp7*_3.2	32364398	32220949	99.56%	15124513	46.94%
si*Ctrl*_1.1	44568787	44142730	99.04%	22009565	49.86%
si*Ctrl*_1.2	42329483	41890625	98.96%	22558102	53.85%
si*Ctrl*_2.1	36631693	36337732	99.20%	15080159	41.50%
si*Ctrl*_2.2	33293677	33002365	99.13%	11379215	34.48%
si*Ctrl*_3.1	73346879	72814388	99.27%	17264291	23.71%
si*Ctrl*_3.2	60957023	60503110	99.26%	15004771	24.80%

Supplementary Table S6. Analysis of SOX2 intensity and SOX2^{+} cell number for 3D images of Ctrl and dKD embryos, related to Fig. 7.

Sample	DNA^{+}	SOX2^{+}	CDX2^{+}	SOX2^{+} (%)	SOX2 intensity	Sample	DNA^{+}	SOX2^{+}	CDX2^{+}	SOX2^{+} (%)	SOX2 intensity
Ctrl-1	57	18	36	31.58	619.46	dKD-1	58	11	43	18.97	728.88
Ctrl-2	71	23	48	32.39	240.94	dKD-2	51	12	36	23.53	418.57
Ctrl-3	57	17	37	29.82	282.49	dKD-3	54	20	37	37.04	752.13
Ctrl-4	61	17	40	27.87	713.76	dKD-4	55	13	31	23.64	794.48
Ctrl-5	61	17	39	27.87	920.04	dKD-5	59	13	36	22.03	560.77
Ctrl-6	57	10	48	17.54	826.12	dKD-6	50	19	26	38.00	959.38
Ctrl-7	57	16	41	28.07	453.99	dKD-7	44	13	23	29.55	758.74
Ctrl-8	73	11	56	15.07	475.78	dKD-8	57	22	30	38.60	1048.90
Ctrl-9	74	14	60	18.92	414.01	dKD-9	51	9	34	17.65	392.49
Ctrl-10	72	9	59	12.50	180.92	dKD-10	50	13	24	26.00	885.42
Ctrl-11	58	14	25	24.14	1164.27	dKD-11	61	15	33	24.59	1036.11
Ctrl-12	58	11	28	18.97	781.62	dKD-12	48	15	25	31.25	565.24
Ctrl-13	63	19	37	30.16	485.43	dKD-13	50	10	23	20.00	1910.94
Ctrl-14	60	24	30	40.00	1645.55	dKD-14	58	17	38	29.31	719.12
Ctrl-15	63	21	32	33.33	693.76	dKD-15	51	14	31	27.45	605.93
Ctrl-16	52	14	30	26.92	1002.50	dKD-16	46	12	32	26.09	973.53
Ctrl-17	57	20	27	35.09	1553.16	dKD-17	50	10	32	20.00	1077.77
Ctrl-18	58	23	34	39.66	1611.83	dKD-18	54	20	38	37.04	451.69
						dKD-19	58	9	48	15.52	645.27

Supplementary Table S7. Analysis of the SOX2 expression of the progeny of *Gfp* or *Rbbp7-Gfp* or *Rbbp7-LincGET-Gfp* overexpressing blastomere at the blastocyst stage, related to Fig. 7.

Cross-sections	GFP cells	SOX2 cells	GFP^+ SOX2 cells	SOX2 cells that are GFP+ (%)	Mean	SD
Gfp-1	45	28	23	82.14	76.21	12.41
Gfp-2	42	15	11	73.33		
Gfp-3	36	18	12	66.67		
Gfp-4	43	10	8	80.00		
Gfp-5	21	3	2	66.67		
Gfp-6	52	10	10	100.00		
Gfp-7	39	9	5	55.56		
Gfp-8	29	17	15	88.24		
Gfp-9	31	15	11	73.33		
Rbbp7-1	52	6	2	33.33	33.89	20.01
Rbbp7-2	15	15	0	0.00		
Rbbp7-3	19	16	2	12.50		
Rbbp7-4	37	6	2	33.33		
Rbbp7-5	28	5	2	40.00		
Rbbp7-6	25	12	2	16.67		
Rbbp7-7	24	15	9	60.00		
Rbbp7-8	16	15	7	46.67		
Rbbp7-9	23	16	10	62.50		
R-L-G-1	13	7	3	42.86	59.91	13.77
R-L-G-2	16	8	5	62.50		
R-L-G-3	14	5	2	40.00		
R-L-G-4	32	14	12	85.71		
R-L-G-5	22	4	3	75.00		
R-L-G-6	29	12	6	50.00		
R-L-G-7	17	6	3	50.00		
R-L-G-8	16	7	5	71.43		
R-L-G-9	47	17	12	70.59		
R-L-G-10	46	19	10	52.63		
R-L-G-11	21	12	7	58.33		

Supplementary Table S8. Primers and oligos used in this study.

Usage	Name	Sequence (5'-3')
RT-qPCR primers	*Rbbp7*-F	GAGCGTGTCATCAACGAAGAG
	Rbbp7-R	CCACTGAACGGTAAGACTGGG
	Sox2-F	GCGGAGTGGAAACTTTTGTCC
	Sox2-R	CGGGAAGCGTGTACTTATCCTT
	Oct4-F	AGAGGATCACCTTGGGGTACA
	Oct4-R	CGAAGCGACAGATGGTGGTC
	Nanog-F	TCTTCCTGGTCCCCACAGTTT
	Nanog-R	GCAAGAATAGTTCTCGGGATGAA
	Cdx2-F	CAAGGACGTGAGCATGTATCC
	Cdx2-R	GTAACCACCGTAGTCCGGGTA
	18S-F	CGCCGCTAGAGGTGAAATTCT
	18S-R	CGAACCTCCGACTTTCGTTCT
	LincGET-F	GTAGAGATGAGGCTTGCTGAGATTTA
	LincGET-R	CCAGCCCTACAAATACTCCTTGTTAA
	Carm1-F	CAGGCTCCAAGTCCAGTAACCTG
	Carm1-R	GGGAGTGGGTGTGATTGACAATC
	Pcbp1-F	GCCTGGCCCAGTATCTAATCAA
	Pcbp1-R	ACGGCTGAACAGAGAATGTGAT
	Id2-F	TCCGGTGAGGTCCGTTAGG
	Id2-R	CAGACTCATCGGGTCGTCC
	Esrrb-F	GGCTCGTCGGACGCCAG
	Esrrb-R	GCATTTGATGGCGGAGTCC
FISH probes	*Rbbp7*-1	/5Biosg/GCATGGGTCATAACCAGGTCATACAGAAACGGTGTAT
	Rbbp7-2	/5Biosg/TTCTGCTCATCAGATGTATGAGTGCCCAGCACTA
	Rbbp7-3	/5Biosg/GGCATATAACGAGCACGATTCACTTCTCCTTCATGGT
	Rbbp7-4	/5Biosg/AGATGCACTCAGGAGATGCCCACTCAGATTAGAATTC
	Rbbp7-5	/5Biosg/CTGATCATCAGCAACAGATCCAAACAAGGACTCATGC
	Rbbp7-6	/5Biosg/CACATTCAGGCGGCGATCAGTACCACTTGAG
	Rbbp7-7	/5Biosg/TGACCCAAGGTTCATTGGGATTCCAGCTGAAGT
	Rbbp7-8	/5Biosg/TCCGATGCCGTGACATCTGACTCTTCATCATTGTAAA
	LincGET-FISH-F	GTAGAGCATGCTCAGTAGAGATGAGG
	LincGET-FISH-R	CCAGCCCTACAAATACTCCTTGTTA
	LincGET-FISH-T7-R	TAATACGACTCACTATAGGGCCAGCCCTACAAATACTCC
siRNA	si*Rbbp7*-1	CCACUGGUCUCCACAUAAU
	si*Rbbp7*-2	AAGUGAAUCGUGCUCGUUA
	si*Rbbp7*-3	AGAUAAGACUGUAGCUUUA
	si*Rbbp7*-4	GAUCCAAUACCACUUCUAA
	si*Ctrl*	UUCUCCGAACGUGUCACGU
ASO	*LincGET*-ASO-1	AAUAUAACACAAACUAGCUG

LincGET-ASO-2	CAUAGAGUUUCUAACUACUC
LincGET-ASO-3	UAUUUAUUUCCAUACCUAGC
LincGET-ASO-4	GAUAGUAAUUAGCUUCCAUC

Supplementary Table legends

- **Supplementary Table S3.** The interacting proteins of RBBP7 identified by IP-MS, related to Fig. 3.
- **Supplementary Table S5.** GO biological process categories of differential H3K9ac modified genes between sister blastomeres of 2CL, related to Fig. 4.
